# Supplementary material for: Prevalence and correlates of long-term e-cigarette and nicotine replacement therapy use: a prospective study in England
Source: BMJ Open. 2019 Oct 11;9(10):e029252. doi: 10.1136/bmjopen-2019-029252 (PMC6797372; doi:10.1136/bmjopen-2019-029252)
Supplement: Author's manuscript [file bmjopen-2019-029252.draft_revisions.pdf]

BMJ Open

BMJ Open is committed to open peer review. As part of this commitment we make the peer review history of every article we publish publicly available.

When an article is published we post the peer reviewers' comments and the authors' responses online. We also post the versions of the paper that were used during peer review. These are the versions that the peer review comments apply to.

The versions of the paper that follow are the versions that were submitted during the peer review process. They are not the versions of record or the final published versions. They should not be cited or distributed as the published version of this manuscript.

BMJ Open is an open access journal and the full, final, typeset and author-corrected version of record of the manuscript is available on our site with no access controls, subscription charges or pay-per-view fees (http://bmjopen.bmj.com).

If you have any questions on BMJ Open's open peer review process please email info.bmjopen@bmj.com

BMJ Open

Long-term e-cigarette and nicotine replacement therapy use in England: a population survey

Journal:	*BMJ Open*
Manuscript ID	bmjopen-2019-029252
Article Type:	Research
Date Submitted by the Author:	18-Jan-2019
Complete List of Authors:	Jackson, Sarah ; University College London, Epidemiology and Public Health;<br>Hill, Emily; University College London, Epidemiology and Public Health<br>Shahab, Lion; UCL, Dept of Epidemiology and Public Health<br>Beard, Emma; UCL,<br>Michie, Susan; University College London, Centre for Outcomes Research and Effectivenes<br>Brown, Jamie; University College London, Psychology & Language Sciences
Keywords:	PUBLIC HEALTH, E-cigarettes, Nicotine replacement therapy, Population-based survey, Long-term use, Smoking cessation

SCHOLARONE™
Manuscripts

Long-term e-cigarette and nicotine replacement therapy use in England: a population survey

Sarah E. Jackson[1], Emily Hill[1], Lion Shahab[1], Emma Beard[1], Susan Michie[2], Jamie Brown[1,2]

[1] Department of Behavioural Science and Health, University College London, London, UK

[2] Department of Clinical, Educational and Health Psychology, University College London, UK

For peer review only

Key words: e-cigarettes; nicotine replacement therapy; population-based survey; long-term use.

Word count: 3,661

Number of tables: 3

Number of figures: 0

Corresponding author: Sarah E Jackson, PhD. Department of Behavioural Science and Health, University College London, 1-19 Torrington Place, London WC1E 6BT

Tel: (44) 207 679 3179

Fax: (44) 207 916 8354

s.e.jackson@ucl.ac.uk

For peer review only - http://bmjopen.bmj.com/site/about/guidelines.xhtml

Abstract

Objectives: To examine the prevalence of, and sociodemographic and smoking-related characteristics associated with, long-term e-cigarette use compared with long-term NRT use.

Design: Cross-sectional and prospective survey.

Setting: England.

Participants: Population-representative sample of 40,933 adults aged 16+.

Main outcome measures: Prevalence of long-term (≥12 months) use of e-cigarettes and NRT by retrospective self-report among baseline respondents (all adults, *n*=40,933; smokers, *n*=8,406), and current use at baseline, 6 months and 12 months in a subsample of smokers who were followed up (*n*=733).

Results: Of baseline respondents, 1.5% (95%CI 1.4-1.6%) of adults and 3.9% (95%CI 3.5-4.3%) of smokers were long-term e-cigarette users and 0.5% (95%CI 0.4-0.6%) of adults and 1.3% (95%CI 1.1-1.5%) of smokers were long-term NRT users. Assessed prospectively, 13.4% (95%CI 10.9-15.9%) of smokers were long-term e-cigarette users and 1.9% (95%CI 0.9-2.9%) were long-term NRT users. Among all adults, long-term use by never smokers of both e-cigarettes (0.1%) and NRT (0.0%) was rare (OR=0.03 and OR=0.02, respectively). Among past-year smokers, long-term e-cigarette and NRT use was higher among older smokers compared with 16-34 year-olds (OR range 1.55-5.21). Long-term e-cigarette use only was lower in smokers who were less educated (OR=0.63, 95%CI 0.49-0.81), from social grades C2DE (OR=0.66, 95%CI 0.52-0.84) and with children in the household (OR=0.66, 95%CI 0.51-0.85). Long-term e-cigarette and NRT use was higher among smokers more motivated to quit (OR=2.05, 95%CI 1.63-2.60 and OR=2.33, 95%CI 1.57-3.46).

Conclusions: In the adult population in England, long-term use of e-cigarettes and NRT is almost exclusively by current or ex-smokers. Only a minority of past-year smokers retrospectively report long-term e-cigarette or NRT use but this figure may be an underestimate, especially for e-cigarette use, which is more than three-fold higher when assessed prospectively.

For peer review only

Strengths and limitations of the study

- Large sample representative of the adult population in England.
- Longitudinal design permitting prospective assessment of long-term use in addition to cross-sectional analyses based on retrospective self-reports.
- Only respondents who reported past-year smoking at baseline were invited to participate in the follow-up surveys, so we were unable to obtain prospective estimates of the prevalence of long-term e-cigarette or NRT use in the entire adult population.
- Attrition bias meant our sample for prospective analyses was older and more socioeconomically advantaged than the group who were lost to follow-up, and more reported recent quitting and long-term use of e-cigarettes or NRT retrospectively at baseline.

For peer review only

For peer review only - http://bmjopen.bmj.com/site/about/guidelines.xhtml

Introduction

Tobacco smoking is one of the leading causes of premature death and disability worldwide (1). The primary cause of smoking-associated morbidity and mortality is the inhalation of toxins produced from the combustion of tobacco (2). Over recent years, electronic cigarettes (e-cigarettes) have rapidly become popular among smokers as a non-combustible alternative to cigarettes that offers safer nicotine delivery (3). But while the prevalence of ever and current use of e-cigarettes has been monitored (e.g., 4–6), there has been little investigation into long-term use of these products. Given an increasing focus on harm reduction in tobacco control, which aims to reduce the harm from combustible products by partial or complete substitution with non-combustible products, high-quality data on long-term use are needed. Understanding who is using e-cigarettes, and for how long, is fundamental in order to evaluate their overall impact on public health.

In England, e-cigarettes are used by around 5% of the adult population (~20% of smokers) (3) and have overtaken nicotine replacement therapy (NRT) as the most popular quitting aid, with over a third of smokers using an e-cigarette in their most recent quit attempt compared with one in five using of NRT (7). Evidence from two randomised controlled trials indicates that using e-cigarettes in a quit attempt increases chances of successful cessation (8). On a population level, the rise in use of e-cigarettes in England and the US has been associated with increases in the overall success rate of quit attempts in the population (9,10), contributing to continued declines in smoking prevalence (11). It is possible that long-term e-cigarette use could help mitigate the high risk of relapse among recent quitters (12); in a survey of US smokers with two-year follow-up, long-term use of e-cigarettes (current use at baseline and follow-up) was associated with four times higher odds of cessation relative to no use (13).

Accumulating evidence demonstrates that using e-cigarettes is substantially less harmful than smoking (3). Toxicology testing has shown that while e-cigarettes can be used to obtain similar levels of nicotine to combustible cigarettes, switching to e-cigarettes can significantly reduce levels of measured carcinogens and toxins relative to smoking only combustible cigarettes, with differences observed within a matter of weeks (14–16). A more favourable toxicity profile has also been observed among long-term e-cigarette users (≥6 months) compared with current cigarette smokers (17). However, surveys have indicated that around half of smokers inaccurately judge e-cigarettes to be more harmful than combustible cigarettes, about as harmful, or are unsure about the relative risk (18), which could discourage use.

For peer review only - http://bmjopen.bmj.com/site/about/guidelines.xhtml

For peer review only

Previous studies that have examined correlates of e-cigarette use have found that smokers who use e-cigarettes tend to be younger than non-users, smoke more heavily, and are more likely to have tried to quit in the past year (4–6). There is also some evidence that e-cigarette use is more prevalent among people with greater socioeconomic advantage (5,6), although the gap appears to have narrowed over recent years (19). However, there is a distinct lack of evidence on both the prevalence of long-term use and the profile of long-term users. This information is important for the evaluation of the overall public health impact of e-cigarettes, which requires specification of a wide variety of parameters beyond the safety of e-cigarettes and their effect on cessation, including the extent and characteristics of people who become long-term users (20).

The present study therefore aimed to examine the prevalence of, and sociodemographic and smoking-related characteristics associated with, long-term (≥12 months) e-cigarette use in England. We also analysed data on long-term NRT use as a case-control, in order to assess the extent to which the prevalence of long-term e-cigarette use and profile of long-term users are specific to e-cigarettes or apply more broadly to non-combustible nicotine products in general. Specifically, we aimed to answer the following research questions:

1. What proportion of adults in England retrospectively report using (i) e-cigarettes or (ii) NRT for at least one year?
2. What proportion of past-year smokers in England retrospectively report using (i) e-cigarettes or (ii) NRT for at least one year?
3. What proportion of past-year smokers in England report current use of (i) e-cigarettes or (ii) NRT at baseline and both 6 and 12-month follow-ups?
4. How do long-term users of e-cigarettes and NRT differ from non-users in their sociodemographic and smoking-related characteristics?

Method

Design and study population

Data were used from the Smoking Toolkit Study (STS), an ongoing monthly repeat cross-sectional survey of adults in England (21). Each month, a form of random location sampling is used to select a new sample of approximately 1,700 adults aged 16 years and older. Comparisons of sociodemographic data and smoking

For peer review only - http://bmjopen.bmj.com/site/about/guidelines.xhtml

prevalence and consumption estimates with national data indicate that STS data are broadly representative of the English population (21). In each wave, respondents complete a face-to-face computer-assisted survey with a trained interviewer. Respondents to the baseline survey between September 2014 and September 2016 who reported smoking in the past year were asked whether they were willing to be re-contacted, and those who agreed were followed up by telephone 6 and 12 months after the baseline interview. For the purpose of the present study, we aggregated data across survey waves. Cross-sectional analyses used data from all adults who responded to the baseline survey during this period, and the prospective analysis used data from respondents who reported past-year smoking at baseline and responded to both the 6 and 12-month follow-up surveys.

Measures

Outcomes: long-term use of e-cigarettes and NRT

The outcomes were long-term (≥12 months) use of e-cigarettes and long-term use of NRT, assessed retrospectively at baseline and prospectively over a 12-month follow-up.

In each of the baseline and follow-up surveys, current use of e-cigarettes and NRT was assessed with three questions that asked whether the respondent used (i) an e-cigarette or other vaping device, or (ii) nicotine replacement therapy (e.g. nicotine patches, gum, spray, or any other product) to help them cut down the amount they smoke, in situations when they are not allowed to smoke, or for any other reason. In the baseline survey, respondents reporting use of either e-cigarettes or NRT were asked how long they had been using this product (less than one week/one to six weeks/more than six weeks and up to 12 weeks/more than 12 weeks and up to 26 weeks/more than 26 weeks and up to 52 weeks/more than 52 weeks).

For the present analyses, long-term use of e-cigarettes/NRT was defined as current use initiated more than 52 weeks prior to the baseline survey for cross-sectional analyses, and as current use at baseline, 6 months and 12 months for prospective analyses.

Sociodemographic and smoking-related characteristics

Data were included on a range of sociodemographic and smoking-related characteristics assessed at baseline.

For peer review only - http://bmjopen.bmj.com/site/about/guidelines.xhtml

Sociodemographic information included: age, sex, ethnicity, region, social grade, level of education, disability, and the presence of children in the household. Ethnicity was categorised as white vs. non-white. Region was defined according to Government Office Region, grouped into three categories: northern, central and southern England. Social grade was categorised as ABC1 (which includes managerial, professional and intermediate occupations) vs. C2DE (which includes small employers and own-account workers, lower supervisory and technical occupations, and semi-routine and routine occupations, never workers and long-term unemployed). Education was categorised as lower (no post-16 qualifications) vs. higher (higher-level qualifications above GCSE level). Disability status was identified from the question "Do you consider yourself to have a disability within the meaning of the Disability Discrimination Act 1995 (yes/no)?". The number of children in the household was self-reported and dichotomised to 0 vs. ≥1.

Smoking-related characteristics included: smoking status, time to first cigarette, consistent motivation to stop and (because it has been shown to be associated with smoking and quitting behaviour (22–24)) excessive alcohol consumption. Smoking status was self-reported by all adults in response to the question: "Which of the following best applies to you (current smoker/stopped in the past year/stopped more than a year ago/never smoked)?". Respondents who reported current smoking or having stopped in the past year ('past-year smokers') were also asked how soon after waking they typically smoked their first cigarette (categorised as within 30 vs. ≥31 minutes; an established indicator of nicotine dependence (25)) and whether they had consistently felt that they wanted to stop in the past year (yes/no). Alcohol consumption was assessed using the Alcohol Use Disorders Identification Test (AUDIT) (26), a 10-item screening tool developed by the World Health Organisation to assess alcohol consumption, drinking behaviours and alcohol dependence, with a score of 8 or more indicating excessive alcohol consumption.

Statistical analysis

The analysis plan and syntax were pre-registered on Open Science Framework (https://osf.io/bpjhk/).

We used chi-square tests to compare the baseline characteristics of individuals who responded to both the 6 and 12-month follow-ups with those who were lost to follow-up in order to assess the representativeness of those followed-up.

We estimated the weighted prevalence of long-term use of (i) e-cigarettes and (ii) NRT in the total adult population at baseline, and in past-year smokers at baseline and over 12-month follow-up. Rim (marginal)

For peer review only

weighting was used to match the English population on the dimensions of age, social grade, region, tenure, ethnicity and working status within sex.

We then used logistic regression to examine the extent to which sociodemographic and smoking-related characteristics were associated with long-term use of e-cigarettes and NRT, assessed at baseline. For each outcome, we analysed bivariate associations with each potential correlate separately, and tested independent associations with a multivariable model that included all variables. We had also intended to analyse associations with long-term use prospectively, but the achieved sample size was lower than we had anticipated, and the prevalence of long-term use was low (particularly for NRT), limiting statistical power.

Results

Long-term use of e-cigarettes and NRT among all adults in England: cross-sectional data

A total of 42,040 adults in England were surveyed between September 2014 and September 2016 and 40,933 (97.4%) were complete cases. The weighted prevalence of long-term e-cigarette use assessed retrospectively among all adults in England was 1.5% (95%CI 1.4-1.6%) and of long-term NRT use was 0.5% (95%CI 0.4-0.6%). Table 1 summarises bivariate and multivariable associations between sociodemographic and smoking-related characteristics and long-term use of e-cigarettes and NRT among all adults in the baseline survey.

In the multivariable model, both long-term e-cigarette use and long-term NRT use were significantly associated with age, region, level of education, disability, and smoking status. Compared with those aged 16-34, long-term e-cigarette use was more prevalent among those aged 35-54 but was not significantly different among those aged ≥55 years. Long-term NRT was significantly more prevalent among those aged 35-54 and ≥55 years. Compared with the north of England, long-term e-cigarette use was less prevalent in central and southern regions, and long-term NRT use was more prevalent in the south. Both long-term e-cigarette use and NRT use were significantly less prevalent among people with no post-16 qualifications and more prevalent among those with a disability. Prevalence of long-term e-cigarette and NRT use did not differ significantly between current smokers and recent ex-smokers, but was significantly less prevalent among never smokers, among whom use of either product was rarely reported (e-cigarettes 0.1%, NRT 0.0%). Long-term NRT use was also significantly less prevalent among long-term ex-smokers, but the

For peer review only - http://bmjopen.bmj.com/site/about/guidelines.xhtml

prevalence of long-term e-cigarette use did not differ significantly between long-term ex-smokers and current smokers.

Long-term NRT use, but not long-term e-cigarette use, was associated with sex and alcohol intake, with higher prevalence observed among women and those who reported excessive alcohol intake. Long-term e-cigarette use, but not long-term NRT use, was associated with the presence of children in the household, with lower prevalence observed among people with children in their household. We observed no significant association between long-term e-cigarette or NRT use and ethnicity or social grade.

Long-term use of e-cigarettes and NRT among past-year smokers in England: cross-sectional data

A total of 8,649 were past-year smokers and 8,406 (97.2%) were complete cases. The weighted prevalence of long-term e-cigarette use assessed retrospectively among past-year smokers in England was 3.9% (95%CI 3.5-4.3%) and of long-term NRT use was 1.3% (95%CI 1.1-1.5%). Table 2 summarises bivariate and multivariable associations between sociodemographic and smoking-related characteristics and long-term use of e-cigarettes and NRT among past-year smokers in the baseline survey.

In the multivariable model, there were significant associations between long-term use of e-cigarettes and NRT and age, region, and motivation to stop smoking, and between long-term use of e-cigarettes and social grade, level of education, and children in the household. Long-term use of e-cigarettes and NRT was more prevalent among older smokers compared with 16-34 year-olds, and among those who were motivated to stop. Compared with the north of England, long-term e-cigarette use was less prevalent in central and southern regions, but long-term NRT use was more prevalent in the south. Long-term e-cigarette use was significantly less prevalent among smokers from social grades C2DE, without post-16 qualifications and with children in their household, while long-term NRT use did not differ significantly according to these factors. We observed no significant association between long-term e-cigarette or NRT use and sex, ethnicity, disability, current smoking status, excessive drinking, or dependence.

Long-term use of e-cigarettes and NRT among all adults in England: prospective data

A total of 733 individuals who reported past-year smoking at baseline completed follow-up surveys at both 6 and 12 months. Characteristics of past-year smokers in the baseline and follow-up samples are summarised in Table 3. Past-year smokers who responded to follow-up were significantly older than those

For peer review only

who did not. A higher proportion of responders were white and fewer were from social grades CD2E or had no post-16 qualifications. More reported a disability and fewer had children in the household. A higher proportion of responders than non-responders were recent ex-smokers and more reported consistent motivation to stop smoking. They were also significantly more likely to report long-term use of e-cigarettes or NRT than those who did not respond to the follow-up surveys. Loss to follow-up was not significantly associated with sex, region, excessive alcohol intake, or dependence. The weighted prevalence of long-term e-cigarette use assessed prospectively among past-year smokers in England was 13.4% (95%CI 10.9-15.9%) and of long-term NRT use was 1.9% (95%CI 0.9-2.9%).

For peer review only

Discussion

In this large, representative sample of adults in England, long-term use of e-cigarettes and NRT was almost exclusively reported by current or ex-smokers. Only a minority of past-year smokers retrospectively reported long-term use of either e-cigarettes (3.9%) or NRT (1.3%) but this figure may be an underestimate: prevalence of current use at three time-points over a 12-month period was substantially higher for both e-cigarettes (13.4%) and NRT (1.9%), although these estimates were likely subject to attrition bias. Both cross-sectionally and prospectively, there was a higher prevalence of long-term e-cigarette use in comparison with NRT use. In adjusted models, long-term use of e-cigarettes and NRT was higher among older smokers and those more motivated to quit smoking. Long-term use of e-cigarettes was less common, and long-term use of NRT was more common, in the south of England compared with the north. Long-term use of e-cigarettes was significantly less prevalent among smokers who were less educated, those from social grades C2DE, and those with children in the household, but these variables were not significantly associated with long-term use of NRT. Neither long-term use of e-cigarettes nor NRT differed significantly according to sex, ethnicity, disability, current smoking status (current vs. recent ex-smokers), excessive alcohol intake, or nicotine dependence.

To our knowledge, this study is the first to examine the prevalence of, and sociodemographic and smoking-related characteristics associated with, long-term use of e-cigarettes. We aimed to identify the prevalence of long-term e-cigarette use cross-sectionally and prospectively, and to contrast usage with long-term NRT use. Our results showed a higher prevalence of long-term e-cigarette use in comparison with NRT. Recent

For peer review only - http://bmjopen.bmj.com/site/about/guidelines.xhtml

prevalence estimates indicate that current use of e-cigarettes is much more popular than NRT (7) and the same appears true for long-term use.

Long-term use of both products was almost exclusively observed among current and former smokers. Concerns have been raised that e-cigarettes may serve as a gateway to cigarette smoking among never-smokers, particularly among youth (27,28), but in our sample, the prevalence of long-term e-cigarette use among never-smokers was just 0.1%, comparable to long-term NRT use. We also observed higher prevalence of long-term e-cigarette use among middle-aged and older adults than in the youngest group (16-34 years).

While the prevalence of long-term use of e-cigarettes and NRT did not differ significantly between current and recent (<1 year) ex-smokers, the relative prevalence of use in long-term (≥1 year) ex-smokers differed between the products. Long-term use of NRT was significantly less prevalent among long-term ex-smokers than current smokers, whereas long-term use of e-cigarettes was similar between these groups. This suggests that people tend to discontinue use of NRT more quickly after quitting smoking than with e-cigarettes.

Insofar that use of alternative nicotine products should promote cessation rather than continued dual use, it is somewhat concerning that long-term use of e-cigarettes was similarly prevalent among current and recent ex-smokers. On the other hand, this appeared equally true for NRT. There have been concerns that dual use of cigarettes and e-cigarettes could reduce the urgency to quit smoking (29) and extend the duration of cigarette smoking (30,31). This would result in a negative overall public health impact, since duration of smoking poses a greater health risk than intensity of smoking (32). However, our results indicate that this is not the case: after mutual adjustment, the recall of long-term use of both e-cigarettes and NRT was higher among smokers who were more motivated to quit. This finding is consistent with previous studies showing that the most common reason for using an e-cigarette is to stop smoking (3), that smokers who use e-cigarettes are more likely to have recently tried to quit (4–6), and that long-term e-cigarette use is associated with a higher rate of smoking cessation (14). It suggests that long-term dual use is not associated with reduced motivation to quit.

Among past-year smokers, long-term use of e-cigarettes specifically was lower among those without post-16 qualifications and those from social grades C2DE. This is consistent with a larger literature on the diffusion of innovation, which recognises the tendency for high status groups to most quickly adopt new ideas and behaviours (33–35); a pattern that was documented for combustible cigarette smoking (36).

For peer review only - http://bmjopen.bmj.com/site/about/guidelines.xhtml
For peer review only

According to this theory, one would expect to see e-cigarette use spread first within high status networks, but patterns of imitation later lead to diffusion of the practice and normative change across classes and down the status hierarchy. The fact that we observed no significant association between education or social grade and long-term NRT use, which has been available for much longer, is consistent with this. With recent evidence indicating that the socioeconomic gradient in e-cigarette use is declining over time (19), we predict that this disparity in long-term use will disappear over the coming years. There were also regional differences, with long-term e-cigarette use more prevalent in the north of England and long-term NRT use more prevalent in the south. The higher prevalence of long-term e-cigarette use in the north is consistent with previous evidence of heavier smoking in the north of England (37) and higher prevalence of e-cigarette use among heavier smokers (4–6).

Prospective analysis of current use at baseline and both 6 and 12-month follow-ups indicated a substantially higher rate of long-term use of both e-cigarettes (13.4%) and NRT (1.9%) among past-year smokers than was seen in the cross-sectional results. While these figures are not directly comparable given the substantial attrition over follow-up assessments and differences in the definition of long-term use (continued use vs. current use at three defined time points), the magnitude of the difference indicates that retrospective recall of how long the products have been used may underestimate what could be expected if users were followed more frequently over time. A study with a greater number of follow-up points over a longer period could offer further insight into this discrepancy.

Strengths of this study include the large, representative sample and prospective design. However, there were several limitations. Only respondents who reported past-year smoking at baseline were invited to participate in the follow-up surveys, so we were unable to obtain prospective estimates of the prevalence of long-term e-cigarette or NRT use in the entire adult population. However, evidence from the cross-sectional results of this study and from previous research (3) suggest that the vast majority of long-term users were current or recent ex-smokers, with low prevalence among never smokers. Another potential issue was attrition bias. Our sample for prospective analyses was older and more socioeconomically advantaged than the group who were lost to follow-up, and more reported long-term use of e-cigarettes or NRT retrospectively at baseline. They were also more likely to have quit recently. This may have contributed to the higher prevalence of long-term use observed in prospective analyses. Finally, we did not consider reasons for or patterns of use. Future studies could build on our findings through more detailed or frequent assessments, and qualitative work with long-term users.

For peer review only - http://bmjopen.bmj.com/site/about/guidelines.xhtml

In conclusion, long-term e-cigarette use is more prevalent than long-term NRT use in the English adult population, but both are almost exclusively by current or ex-smokers. The profile of long-term e-cigarette users is broadly similar to that of long-term NRT users, although there are some sociodemographic and regional differences between the two, with long-term e-cigarette use more prevalent among smokers with greater socioeconomic advantage and in the north of England, and long-term NRT use more prevalent among smokers in the south. Prospective assessment of long-term use produces substantially higher estimates of prevalence, particularly for e-cigarettes, than retrospective recall, although this may to some extent be accounted for by differences in the sample and definitions used. These results add to the descriptive picture of e-cigarette use in England, providing novel insight into long-term use. This information can be incorporated into broader evaluations of population-level use of e-cigarettes and their potential impact on public health.

For peer review only

For peer review only - http://bmjopen.bmj.com/site/about/guidelines.xhtml

References

1. World Health Organization. Tobacco [Internet]. 2018 [cited 2018 Dec 2]. Available from: http://www.who.int/news-room/fact-sheets/detail/tobacco

2. Royal College of Physicians. Harm reduction in nicotine addiction: helping people who can't quit [Internet]. 2007 [cited 2018 Dec 5]. Available from: https://shop.rcplondon.ac.uk/products/harm-reduction-in-nicotine-addiction-helping-people-who-cant-quit

3. McNeill A, Brose LS, Calder R, Bauld L, Robson D. Evidence review of e-cigarettes and heated tobacco products 2018. A report commissioned by Public Health England. [Internet]. 2018 [cited 2018 Jun 21]. Available from: https://www.gov.uk/government/publications/e-cigarettes-and-heated-tobacco-products-evidence-review/evidence-review-of-e-cigarettes-and-heated-tobacco-products-2018-executive-summary

4. Vardavas CI, Filippidis FT, Agaku IT. Determinants and prevalence of e-cigarette use throughout the European Union: a secondary analysis of 26 566 youth and adults from 27 Countries. Tob Control. 2015 Sep 1;24(5):442–8.

5. Levy DT, Yuan Z, Li Y. The Prevalence and Characteristics of E-Cigarette Users in the U.S. Int J Environ Res Public Health [Internet]. 2017 Oct [cited 2018 Dec 5];14(10). Available from: https://www.ncbi.nlm.nih.gov/pmc/articles/PMC5664701/

6. Brown J, West R, Beard E, Michie S, Shahab L, McNeill A. Prevalence and characteristics of e-cigarette users in Great Britain: Findings from a general population survey of smokers. Addict Behav. 2014 Jun 1;39(6):1120–5.

7. Smoking in England. Electronic cigarettes in England - latest trends [Internet]. 2018 [cited 2018 Oct 25]. Available from: http://www.smokinginengland.info/latest-statistics/

8. McRobbie H, Bullen C, Hartmann-Boyce J, Hajek P. Electronic cigarettes for smoking cessation and reduction. Cochrane Database Syst Rev. 2014;(12):CD010216.

9. Beard E, West R, Michie S, Brown J. Association between electronic cigarette use and changes in quit attempts, success of quit attempts, use of smoking cessation pharmacotherapy, and use of stop smoking services in England: time series analysis of population trends. BMJ. 2016 Sep 13;354:i4645.

10. Zhu S-H, Zhuang Y-L, Wong S, Cummins SE, Tedeschi GJ. E-cigarette use and associated changes in population smoking cessation: evidence from US current population surveys. BMJ. 2017 Jul 26;358:j3262.

11. Royal College of Physicians. Nicotine without smoke: Tobacco harm reduction [Internet]. 2016 Apr [cited 2018 Dec 5]. Available from: https://www.rcplondon.ac.uk/projects/outputs/nicotine-without-smoke-tobacco-harm-reduction-0

12. Hughes JR, Keely J, Naud S. Shape of the relapse curve and long-term abstinence among untreated smokers. Addict Abingdon Engl. 2004 Jan;99(1):29–38.

For peer review only - http://bmjopen.bmj.com/site/about/guidelines.xhtml

13. Zhuang Y-L, Cummins SE, Sun JY, Zhu S-H. Long-term e-cigarette use and smoking cessation: a longitudinal study with US population. Tob Control. 2016 Oct 1;25(Suppl 1):i90–5.

14. Goniewicz ML, Gawron M, Smith DM, Peng M, Jacob P, Benowitz NL. Exposure to Nicotine and Selected Toxicants in Cigarette Smokers Who Switched to Electronic Cigarettes: A Longitudinal Within-Subjects Observational Study. Nicotine Tob Res. 2017 Feb 1;19(2):160–7.

15. McRobbie H, Phillips A, Goniewicz ML, Smith KM, Knight-West O, Przulj D, et al. Effects of Switching to Electronic Cigarettes with and without Concurrent Smoking on Exposure to Nicotine, Carbon Monoxide, and Acrolein. Cancer Prev Res Phila Pa. 2015 Sep;8(9):873–8.

16. Hecht SS, Carmella SG, Kotandeniya D, Pillsbury ME, Chen M, Ransom BWS, et al. Evaluation of toxicant and carcinogen metabolites in the urine of e-cigarette users versus cigarette smokers. Nicotine Tob Res Off J Soc Res Nicotine Tob. 2015 Jun;17(6):704–9.

17. Shahab L, Goniewicz ML, Blount BC, Brown J, McNeill A, Alwis KU, et al. Nicotine, Carcinogen, and Toxin Exposure in Long-Term E-Cigarette and Nicotine Replacement Therapy Users: A Cross-sectional Study. Ann Intern Med. 2017 Mar 21;166(6):390–400.

18. Wilson S, Partos T, McNeill A, Brose LS. Harm perceptions of e-cigarettes and other nicotine products in a UK sample. Addict Abingdon Engl. 2019 Jan 3;

19. Kock L, Shahab L, West R, Brown J. E-cigarette use in England 2014-17 as a function of socio-economic profile. Addict Abingdon Engl. 2018 Oct 10;

20. Levy DT, Cummings KM, Villanti AC, Niaura R, Abrams DB, Fong GT, et al. A framework for evaluating the public health impact of e-cigarettes and other vaporized nicotine products. Addict Abingdon Engl. 2017;112(1):8–17.

21. Fidler JA, Shahab L, West O, Jarvis MJ, McEwen A, Stapleton JA, et al. “The smoking toolkit study”: a national study of smoking and smoking cessation in England. BMC Public Health. 2011 Jun 18;11:479.

22. Falk DE, Yi H, Hiller-Sturmhöfel S. An epidemiologic analysis of co-occurring alcohol and tobacco use and disorders: findings from the National Epidemiologic Survey on Alcohol and Related Conditions. Alcohol Res Health J Natl Inst Alcohol Abuse Alcohol. 2006;29(3):162–71.

23. Beard E, West R, Michie S, Brown J. Association between smoking and alcohol-related behaviours: a time–series analysis of population trends in England. Addict Abingdon Engl. 2017 Oct;112(10):1832–41.

24. Brown J, West R, Beard E, Brennan A, Drummond C, Gillespie D, et al. Are recent attempts to quit smoking associated with reduced drinking in England? A cross-sectional population survey. BMC Public Health. 2016 Jul 22;16(1):535.

25. Heatherton TF, Kozlowski LT, Frecker RC, FAGERSTROM K-O. The Fagerström test for nicotine dependence: a revision of the Fagerstrom Tolerance Questionnaire. Br J Addict. 1991;86(9):1119–1127.

26. Babor TF, Higgins-Biddle JC, Saunders JB, Monteiro MG, Organization WH. AUDIT: the alcohol use disorders identification test: guidelines for use in primary health care. 2001;

For peer review only

For peer review only - http://bmjopen.bmj.com/site/about/guidelines.xhtml

27. Soneji S, Barrington-Trimis JL, Wills TA, Leventhal AM, Unger JB, Gibson LA, et al. Association Between Initial Use of e-Cigarettes and Subsequent Cigarette Smoking Among Adolescents and Young Adults: A Systematic Review and Meta-analysis. JAMA Pediatr. 2017 Aug 1;171(8):788–97.

28. Schneider S, Diehl K. Vaping as a Catalyst for Smoking? An Initial Model on the Initiation of Electronic Cigarette Use and the Transition to Tobacco Smoking Among Adolescents. Nicotine Tob Res Off J Soc Res Nicotine Tob. 2016 May;18(5):647–53.

29. Grana R, Benowitz N, Glantz SA. E-cigarettes: a scientific review. Circulation. 2014 May 13;129(19):1972–86.

30. Furlow B. US Government warns against long-term dual use of conventional and e-cigarettes. Lancet Respir Med. 2015 May;3(5):345.

31. Maziak W. Harm reduction at the crossroads: the case of e-cigarettes. Am J Prev Med. 2014 Oct;47(4):505–7.

32. Doll R, Peto R. Cigarette smoking and bronchial carcinoma: dose and time relationships among regular smokers and lifelong non-smokers. J Epidemiol Community Health. 1978 Dec 1;32(4):303–13.

33. Strang D, Soule SA. Diffusion in Organizations and Social Movements: From Hybrid Corn to Poison Pills. Annu Rev Sociol. 1998;24(1):265–90.

34. Rogers EM. Diffusion of Innovations, 4th Edition. Simon and Schuster; 2010. 550 p.

35. Katz E. Theorizing Diffusion: Tarde and Sorokin Revisited. Ann Am Acad Pol Soc Sci. 1999 Nov 1;566(1):144–55.

36. Ferrence R. Deadly Fashion: The Rise and Fall of Cigarette Smoking in North America. 1 edition. New York: Garland Science; 1990. 167 p.

37. Beard E, Brown J, West R, Angus C, Kaner E, Michie S. Healthier central England or North–South divide? Analysis of national survey data on smoking and high-risk drinking. BMJ Open. 2017 Mar 1;7(3):e014210.

For peer review only

Declarations

Ethics approval and consent to participate

Ethical approval for the Smoking Toolkit Study was granted originally by the UCL Ethics Committee (ID 0498/001) and participants provided full informed consent. The data are not collected by UCL and are anonymised when received by UCL.

Availability of data and materials

The datasets used and/or analysed during the current study are available from the corresponding author on reasonable request.

Competing interests

JB received unrestricted research funding from Pfizer, who manufacture smoking cessation medications. LS has received honoraria for talks, an unrestricted research grant and travel expenses to attend meetings and workshops from Pfizer, and has acted as paid reviewer for grant awarding bodies and as a paid consultant for health care companies. All authors declare no financial links with tobacco companies or e-cigarette manufacturers or their representatives.

Funding

This work was supported by Cancer Research UK, grant numbers C1417/A22962 and C44576/A19501. The funders had no final role in the study design; in the collection, analysis and interpretation of data; in the writing of the report; or in the decision to submit the paper for publication. All researchers listed as authors are independent from the funders and all final decisions about the research were taken by the investigators and were unrestricted.

Author contributions

JB and EH conceived of the study. SEJ, EH and JB analysed and interpreted the data. SEJ and EH drafted the manuscript. All authors critically revised the manuscript for important intellectual content.

For peer review only - http://bmjopen.bmj.com/site/about/guidelines.xhtml

Table 1 Factors associated with long-term use of e-cigarettes or NRT by adults in England: cross-sectional data (*n*=40,933)

	E-cigarettes			NRT		
	**% (*n*) long-term use**	**OR [95% CI]<br>*p***	**Adj OR [95% CI]<br>*p***	**% (*n*) long-term use**	**OR [95% CI]<br>*p***	**Adj OR [95% CI]<br>*p***
All adults*	1.5 (604)	-	-	0.5 (205)	-	-
Age in years						
16-34	1.1 (138)	1.00	1.00	0.2 (21)	1.00	1.00
35-54	2.1 (252)	1.89 (1.53:2.33)<br><0.001	1.71 (1.38:2.13)<br><0.001	0.7 (87)	4.26 (2.65:6.87)<br><0.001	3.98 (2.45:6.47)<br><0.001
≥55	1.1 (185)	1.01 (0.81:1.26)<br>0.912	0.78 (0.60:1.02)<br>0.064	0.6 (89)	3.21 (2.00:5.17)<br><0.001	3.25 (1.91:5.52)<br><0.001
Sex						
Men	1.5 (308)	1.00	1.00	0.5 (94)	1.00	1.00
Women	1.3 (267)	0.90 (0.76:1.06)<br>0.190	1.04 (0.87:1.23)<br>0.683	0.5 (103)	1.14 (0.86:1.50)<br>0.377	1.39 (1.04:1.86)<br>0.025
Ethnicity						
Non-white	0.6 (42)	1.00	1.00	0.1 (10)	1.00	1.00
White	1.6 (533)	2.52 (1.84:3.46)<br><0.001	1.12 (0.80:1.56)<br>0.510	0.5 (187)	3.69 (1.95:6.98)<br><0.001	1.48 (0.77:2.86)<br>0.240
Social grade						
ABC1	1.3 (292)	1.00	1.00	0.5 (102)	1.00	1.00
C2DE	1.5 (283)	1.12 (0.95:1.32)<br>0.191	0.91 (0.76:1.10)<br>0.326	0.5 (95)	1.07 (0.81:1.42)<br>0.629	0.93 (0.68:1.27)<br>0.644
Region						
North	2.1 (281)	1.00	1.00	0.4 (57)	1.00	1.00
Central	1.1 (132)	0.51 (0.41:0.63)<br><0.001	0.60 (0.48:0.74)<br><0.001	0.5 (59)	1.13 (0.79:1.63)<br>0.507	1.41 (0.97:2.04)<br>0.069
South	1.0 (162)	0.47 (0.39:0.57)<br><0.001	0.55 (0.45:0.67)<br><0.001	0.5 (81)	1.18 (0.84:1.66)<br>0.343	1.45 (1.02:2.04)<br>0.036
Level of education						
Post-16 qualifications	1.4 (371)	1.00	1.00	0.5 (126)	1.00	1.00
No post-16 qualifications	1.4 (204)	0.95 (0.80:1.13)<br>0.569	0.79 (0.65:0.95)<br>0.012	0.5 (71)	0.98 (0.73:1.31)<br>0.867	0.71 (0.52:0.98)<br>0.036
Disability						
No	1.3 (463)	1.00	1.00	0.4 (152)	1.00	1.00
Yes	2.3 (112)	1.83 (1.49:2.26)<br><0.001	1.34 (1.07:1.66)<br>0.009	0.9 (45)	2.23 (1.60:3.12)<br><0.001	1.52 (1.08:2.15)<br>0.017
Children in the household						
0	1.4 (413)	1.00	1.00	0.5 (145)	1.00	1.00
≥1	1.4 (162)	0.96 (0.80:1.15)<br>0.660	0.80 (0.65:0.98)<br>0.030	0.4 (52)	0.88 (0.64:1.21)<br>0.420	1.00 (0.69:1.45)<br>0.998
Current smoking status						
Current smoker	3.8 (302)	1.00	1.00	1.4 (107)	1.00	1.00
Recent (<1y) ex-smoker	3.8 (21)	0.98 (0.63:1.54)<br>0.944	0.93 (0.59:1.46)<br>0.747	1.3 (7)	0.93 (0.43:2.00)<br>0.843	0.87 (0.40:1.89)<br>0.724
Long-term (≥1y) ex-smoker	3.2 (225)	0.83 (0.70:1.00)<br>0.043	0.90 (0.74:1.09)<br>0.274	1.1 (76)	0.80 (0.59:1.07)<br>0.136	0.62 (0.45:0.85)<br>0.003

For peer review only

For peer review only - http://bmjopen.bmj.com/site/about/guidelines.xhtml

Never smoker	0.1 (27)	0.03 (0.02:0.04) <0.001	0.03 (0.02:0.04) <0.001	0.0 (7)	0.02 (0.01:0.04) <0.001	0.02 (0.01:0.04) <0.001
Excessive alcohol intake						
No	1.3 (449)	1.00	1.00	0.4 (149)	1.00	1.00
Yes	2.4 (126)	1.95 (1.60:2.39) <0.001	1.02 (0.83:1.27) 0.830	0.9 (48)	2.22 (1.61:3.09) <0.001	1.60 (1.13:2.26) 0.008

The adjusted model includes all variables in the table and year of survey.

*This figure is weighted, and therefore the effective N does not correspond precisely with the unweighted figures reported elsewhere in the table.

OR = odds ratio; CI = confidence interval; SD = standard deviation; NRT = nicotine replacement therapy.

For peer review only

Table 2 Factors associated with long-term use of e-cigarettes or NRT by past-year smokers in England: cross-sectional data (*n*=8,406)

	E-cigarettes			NRT		
	**% (*n*) long-term use**	**OR [95% CI] *p***	**Adj OR [95% CI] *p***	**% (*n*) long-term use**	**OR [95% CI] *p***	**Adj OR [95% CI] *p***
All adults*	3.9 (327)	-	-	1.3 (112)	-	-
Age in years						
16-34	2.6 (88)	1.00	1.00	0.5 (15)	1.00	1.00
35-54	4.9 (135)	1.88 (1.43:2.47) <0.001	1.90 (1.44:2.52) <0.001	1.7 (46)	3.72 (2.07:6.67) <0.001	3.47 (1.92:6.27) <0.001
≥55	4.3 (98)	1.64 (1.22:2.19) 0.001	1.55 (1.12:2.13) 0.008	2.3 (52)	5.10 (2.86:9.08) <0.001	5.21 (2.79:9.72) <0.001
Sex						
Men	1.3 (57)	1.00	1.00	1.3 (57)	1.00	1.00
Women	1.5 (56)	0.96 (0.77:1.20) 0.702	1.00 (0.80:1.27) 0.974	1.4 (56)	1.15 (0.79:1.66) 0.474	1.14 (0.78:1.68) 0.498
Ethnicity						
Non-white	3.4 (31)	1.00	1.00	0.9 (8)	1.00	1.00
White	3.9 (290)	1.15 (0.79:1.68) 0.460	1.01 (0.68:1.50) 0.961	1.4 (105)	1.62 (0.79:3.33) 0.192	1.33 (0.63:2.80) 0.459
Social grade						
ABC1	5.2 (167)	1.00	1.00		1.00	1.00
C2DE	3.0 (154)	0.56 (0.45:0.70) <0.001	0.66 (0.52:0.84) 0.001	1.1 (58)	0.65 (0.45:0.94) 0.022	0.71 (0.47:1.06) 0.091
Region						
North	4.7 (147)	1.00	1.00	1.0 (32)	1.00	1.00
Central	3.4 (81)	0.73 (0.55:0.96) 0.023	0.79 (0.59:1.04) 0.094	1.4 (33)	1.38 (0.85:2.25) 0.197	1.52 (0.92:2.50) 0.100
South	3.2 (93)	0.68 (0.52:0.88) 0.004	0.68 (0.52:0.90) 0.006	1.7 (48)	1.64 (1.05:2.58) 0.030	1.76 (1.11:2.79) 0.016
Level of education						
Post-16 qualifications	4.7 (214)	1.00	1.00	1.4 (65)	1.00	1.00
No post-16 qualifications	2.7 (107)	0.57 (0.45:0.72) <0.001	0.63 (0.49:0.81) <0.001	1.2 (48)	0.85 (0.59:1.24) 0.411	0.85 (0.56:1.27) 0.426
Disability						
No	3.7 (260)	1.00	1.00	1.2 (86)	1.00	1.00
Yes	4.7 (61)	1.29 (0.97:1.72) 0.079	1.22 (0.90:1.64) 0.197	2.1 (27)	1.73 (1.12:2.67) 0.014	1.46 (0.93:2.29) 0.103
Children in the household						
0	4.3 (240)	1.00	1.00	1.5 (83)	1.00	1.00
≥1	2.9 (81)	0.66 (0.51:0.85) 0.001	0.68 (0.51:0.90) 0.007	1.1 (30)	0.71 (0.47:1.08) 0.111	1.07 (0.66:1.72) 0.795
Current smoking status						
Current smoker	3.8 (301)	1.00	1.00	1.3 (106)	1.00	1.00
Recent (<1y) ex-smoker	3.7 (20)	0.96 (0.61:1.52) 0.858	0.69 (0.43:1.11) 0.124	1.3 (7)	0.95 (0.44:2.06) 0.904	0.69 (0.32:1.52) 0.361
Excessive alcohol intake						

For peer review only

No	3.7 (242)	1.00	1.00	1.3 (84)	1.00	1.00
Yes	4.2 (79)	1.14 (0.88:1.48) 0.323	1.02 (0.78:1.35) 0.871	1.5 (29)	1.20 (0.79:1.84) 0.396	1.52 (0.97:2.38) 0.065
Time to first cigarette						
31 or more minutes	3.8 (163)	1.00	1.00	1.2 (53)	1.00	1.00
Within 30 minutes	3.9 (158)	1.02 (0.82:1.28) 0.833	1.07 (0.85:1.35) 0.581	1.5 (60)	1.20 (0.83:1.74) 0.341	1.16 (0.79:1.70) 0.447
Consistent motivation to stop						
No	2.6 (123)	1.00	1.00	0.9 (41)	1.00	1.00
Yes	5.4 (198)	2.11 (1.68:2.66) <0.001	2.05 (1.63:2.60) <0.001	1.9 (72)	2.27 (1.54:3.33) <0.001	2.33 (1.57:3.46) <0.001

The adjusted model includes all variables in the table and year of survey.

*This figure is weighted, and therefore the effective N does not correspond precisely with the unweighted figures reported elsewhere in the table.

OR = odds ratio; CI = confidence interval; SD = standard deviation; NRT = nicotine replacement therapy.

For peer review only - http://bmjopen.bmj.com/site/about/guidelines.xhtml

Table 3 Comparison of the baseline and follow-up samples of past-year smokers

	Baseline sample* % (*n*=8,406)	Follow-up sample % (*n*=733)	*p***
Age in years			
16-34	39.6 (3326)	15.6 (114)	<0.001
35-54	33.0 (2777)	34.4 (252)	-
≥55	27.4 (2303)	50.1 (367)	-
Women	46.2 (3885)	44.9 (329)	0.449
White ethnicity	89.1 (7488)	95.4 (699)	<0.001
Social grade C2DE	61.8 (5193)	51.3 (376)	<0.001
Region			
North	37.5 (3150)	39.4 (289)	0.137
Central	28.1 (2363)	25.0 (183)	-
South	34.4 (2893)	35.6 (261)	-
No post-16 qualifications	46.3 (3893)	40.4 (296)	0.001
Has a disability	15.5 (1304)	22.6 (166)	<0.001
≥1 children in the household	33.6 (2826)	22.9 (168)	<0.001
Current smoking status			
Current smoker	93.5 (7862)	91.7 (672)	0.033
Recent (<1y) ex-smoker	6.5 (544)	8.3 (61)	-
Excessive alcohol intake	22.4 (1879)	20.1 (147)	0.118
First cigarette within 30 minutes	48.6 (4089)	50.3 (369)	0.336
Consistent motivation to stop	43.9 (3694)	48.6 (356)	0.008
Long-term e-cigarette use***	3.8 (321)	5.5 (40)	0.015
Long-term NRT use***	1.3 (113)	2.6 (19)	0.002

*Past-year smokers only.
**Comparison of respondents who did and did not provide follow-up data.
***Assessed at baseline.

For peer review only

STROBE Statement—Checklist of items that should be included in reports of ***cross-sectional studies***

	Item No	Recommendation	Page
**Title and abstract**	1	(*a*) Indicate the study's design with a commonly used term in the title or the abstract	1
		(*b*) Provide in the abstract an informative and balanced summary of what was done and what was found	2
**Introduction**			
Background/rationale	2	Explain the scientific background and rationale for the investigation being reported	4
Objectives	3	State specific objectives, including any prespecified hypotheses	5
**Methods**			
Study design	4	Present key elements of study design early in the paper	5
Setting	5	Describe the setting, locations, and relevant dates, including periods of recruitment, exposure, follow-up, and data collection	6
Participants	6	(*a*) Give the eligibility criteria, and the sources and methods of selection of participants	6
Variables	7	Clearly define all outcomes, exposures, predictors, potential confounders, and effect modifiers. Give diagnostic criteria, if applicable	6/7
Data sources/ measurement	8*	For each variable of interest, give sources of data and details of methods of assessment (measurement). Describe comparability of assessment methods if there is more than one group	6
Bias	9	Describe any efforts to address potential sources of bias	7
Study size	10	Explain how the study size was arrived at	8
Quantitative variables	11	Explain how quantitative variables were handled in the analyses. If applicable, describe which groupings were chosen and why	6/7
Statistical methods	12	(*a*) Describe all statistical methods, including those used to control for confounding	7/8
		(*b*) Describe any methods used to examine subgroups and interactions	7/8
		(*c*) Explain how missing data were addressed	8
		(*d*) If applicable, describe analytical methods taking account of sampling strategy	7/8
		(*e*) Describe any sensitivity analyses	na
**Results**			
Participants	13*	(a) Report numbers of individuals at each stage of study—eg numbers potentially eligible, examined for eligibility, confirmed eligible, included in the study, completing follow-up, and analysed	8
		(b) Give reasons for non-participation at each stage	8
		(c) Consider use of a flow diagram	na
Descriptive data	14*	(a) Give characteristics of study participants (eg demographic, clinical, social) and information on exposures and potential confounders	8/9
		(b) Indicate number of participants with missing data for each variable of interest	na
Outcome data	15*	Report numbers of outcome events or summary measures	Tables
Main results	16	(*a*) Give unadjusted estimates and, if applicable, confounder-adjusted estimates and their precision (eg, 95% confidence interval). Make clear which confounders were adjusted for and why they were included	Tables

For peer review only - http://bmjopen.bmj.com/site/about/guidelines.xhtml

		(*b*) Report category boundaries when continuous variables were categorized	8
		(*c*) If relevant, consider translating estimates of relative risk into absolute risk for a meaningful time period	na
Other analyses	17	Report other analyses done—eg analyses of subgroups and interactions, and sensitivity analyses	na
**Discussion**			
Key results	18	Summarise key results with reference to study objectives	10
Limitations	19	Discuss limitations of the study, taking into account sources of potential bias or imprecision. Discuss both direction and magnitude of any potential bias	12
Interpretation	20	Give a cautious overall interpretation of results considering objectives, limitations, multiplicity of analyses, results from similar studies, and other relevant evidence	13
Generalisability	21	Discuss the generalisability (external validity) of the study results	12
**Other information**			
Funding	22	Give the source of funding and the role of the funders for the present study and, if applicable, for the original study on which the present article is based	17

*Give information separately for exposed and unexposed groups.

Note: An Explanation and Elaboration article discusses each checklist item and gives methodological background and published examples of transparent reporting. The STROBE checklist is best used in conjunction with this article (freely available on the Web sites of PLoS Medicine at http://www.plosmedicine.org/, Annals of Internal Medicine at http://www.annals.org/, and Epidemiology at http://www.epidem.com/). Information on the STROBE Initiative is available at www.strobe-statement.org.

For peer review only

For peer review only - http://bmjopen.bmj.com/site/about/guidelines.xhtml

BMJ Open

Prevalence and correlates of long-term e-cigarette and nicotine replacement therapy use: a prospective study in England

Journal:	*BMJ Open*
Manuscript ID	bmjopen-2019-029252.R1
Article Type:	Original research
Date Submitted by the Author:	15-Jun-2019
Complete List of Authors:	Jackson, Sarah ; University College London, Epidemiology and Public Health;<br>Hill, Emily; University College London, Epidemiology and Public Health<br>Shahab, Lion; UCL, Dept of Epidemiology and Public Health<br>Beard, Emma; UCL,<br>Michie, Susan; University College London, Centre for Outcomes Research and Effectivenes<br>Brown, Jamie; University College London, Psychology & Language Sciences
**Primary Subject Heading**:	Smoking and tobacco
Secondary Subject Heading:	Addiction, Public health
Keywords:	PUBLIC HEALTH, E-cigarettes, Nicotine replacement therapy, Population-based survey, Long-term use, Smoking cessation

SCHOLARONE™
Manuscripts

Prevalence and correlates of long-term e-cigarette and nicotine replacement therapy use: a prospective study in England

Sarah E. Jackson[1], Emily Hill[1], Lion Shahab[1], Emma Beard[1], Susan Michie[2], Jamie Brown[1,2]

[1] Department of Behavioural Science and Health, University College London, London, UK

[2] Department of Clinical, Educational and Health Psychology, University College London, UK

Key words: e-cigarettes; nicotine replacement therapy; population-based survey; long-term use.

Word count: 4450

Number of tables: 3

Number of figures: 0

Corresponding author: Sarah E Jackson, PhD. Department of Behavioural Science and Health, University College London, 1-19 Torrington Place, London WC1E 6BT

Tel: (44) 207 679 3179

Fax: (44) 207 916 8354

s.e.jackson@ucl.ac.uk

For peer review only

Abstract

Objectives: To examine the prevalence of, and sociodemographic and smoking-related characteristics associated with, long-term e-cigarette use compared with long-term NRT use.

Design: Cross-sectional and prospective survey, the Smoking Toolkit Study, with baseline data collected between September 2014 and September 2016 and follow-ups at 6 and 12 months.

Setting: England.

Participants: Population-representative sample of 40,933 adults aged 16+.

Main outcome measures: Prevalence of long-term (≥12 months) use of e-cigarettes and NRT by retrospective self-report among baseline respondents (all adults, *n*=40,933; smokers, *n*=8,406), and current use at baseline, 6 months and 12 months in a subsample of smokers who responded to follow-up (*n*=733).

Results: Of baseline respondents, 1.5% (95%CI=1.4-1.6%, *n*=604) of adults and 3.9% (95%CI=3.5-4.3%, *n*=327) of smokers were long-term e-cigarette users and 0.5% (95%CI=0.4-0.6%, *n*=205) of adults and 1.3% (95%CI=1.1-1.5%, *n*=112) of smokers were long-term NRT users. Assessed prospectively, 13.4% (95%CI=10.9-15.9%, *n*=100) of smokers were long-term e-cigarette users and 1.9% (95%CI=0.9-2.9%, *n*=14) were long-term NRT users. Among all adults, long-term use by never smokers of either e-cigarettes (0.1%, *n*=27) or NRT (0.0%, *n*=7) was rare. Among past-year smokers, long-term e-cigarette and NRT use was higher among older smokers compared with 16-34 year-olds (OR range=1.55-5.21). Long-term e-cigarette use only was lower in smokers who were less educated (OR=0.63, 95%CI=0.49-0.81), from social grades C2DE (OR=0.66, 95%CI 0.52-0.84) and with children in the household (OR=0.66, 95%CI=0.51-0.85). Long-term e-cigarette use and long-term NRT use were higher among smokers more motivated to quit (OR=2.05, 95%CI=1.63-2.60 and OR=2.33, 95%CI=1.57-3.46).

Conclusions: In the adult population in England, long-term use of e-cigarettes and long-term use of NRT are almost exclusively by current or ex-smokers. Only a minority of past-year smokers retrospectively report long-term e-cigarette or NRT use but this figure may be an underestimate, especially for e-cigarette use, which is more than three-fold higher when assessed prospectively.

For peer review only

For peer review only - http://bmjopen.bmj.com/site/about/guidelines.xhtml

Strengths and limitations of the study

- Large sample representative of the adult population in England.
- Longitudinal design permitting prospective assessment of long-term use in addition to cross-sectional analyses based on retrospective self-reports.
- Only respondents who reported past-year smoking at baseline were invited to participate in the follow-up surveys, so we were unable to obtain prospective estimates of the prevalence of long-term e-cigarette or NRT use in the entire adult population.
- Substantial attrition bias meant our sample for prospective analyses was older and more socioeconomically advantaged than the group who were lost to follow-up, and more reported recent quitting and long-term use of e-cigarettes or NRT retrospectively at baseline.

For peer review only

For peer review only - http://bmjopen.bmj.com/site/about/guidelines.xhtml

Introduction

Tobacco smoking is one of the leading causes of premature death and disability worldwide (1). The primary cause of smoking-associated morbidity and mortality is the inhalation of toxins produced from the combustion of tobacco (2). Over recent years, electronic cigarettes (e-cigarettes) have rapidly become popular among smokers as a non-combustible alternative to cigarettes that offers safer nicotine delivery (3). But while the prevalence of ever and current use of e-cigarettes has been monitored (e.g., 4–6), there has been little investigation into long-term use of these products. Given an increasing focus on harm reduction in tobacco control, which aims to reduce the harm from combustible products by partial or complete substitution with non-combustible products, high-quality data on long-term use are needed. Understanding who is using e-cigarettes, and for how long, is fundamental in order to evaluate their overall impact on public health.

In England, e-cigarettes are used by around 5% of the adult population (~20% of smokers) (3) and have overtaken nicotine replacement therapy (NRT) as the most popular quitting aid, with over a third of smokers using an e-cigarette in their most recent quit attempt compared with one in five using of NRT (7). In England, e-cigarettes are not currently available on prescription but are subject to the EU Tobacco Products Directive (including advertising restrictions) and Trading Standards and can be bought online and from vape shops, pharmacies and other retail outlets, while NRT can be bought over the counter or obtained on prescription from a licensed health professional. Evidence from three randomised controlled trials indicates that using e-cigarettes in a quit attempt increases chances of successful cessation (8,9). On a population level, the rise in use of e-cigarettes in England and the US has been associated with increases in the overall success rate of quit attempts in the population (10,11), likely contributing to continued declines in smoking prevalence (12). It is possible that long-term e-cigarette use could help mitigate the high risk of relapse among recent quitters (13); in a survey of US smokers with two-year follow-up, long-term use of e-cigarettes (current use at baseline and follow-up) was associated with four times higher odds of cessation relative to no use (14).

Accumulating evidence demonstrates that using e-cigarettes is substantially less harmful than smoking (3). Toxicology testing has shown that while e-cigarettes can be used to obtain similar levels of nicotine to combustible cigarettes, switching to e-cigarettes can significantly reduce levels of measured carcinogens and toxins relative to smoking only combustible cigarettes, with differences observed within a matter of weeks (15–17). A more favourable toxicity profile has also been observed among long-term e-cigarette

For peer review only - http://bmjopen.bmj.com/site/about/guidelines.xhtml

users (≥6 months) compared with current cigarette smokers (18). However, surveys have indicated that around half of smokers inaccurately judge e-cigarettes to be more harmful than combustible cigarettes, about as harmful, or are unsure about the relative risk (19), which could discourage use.

Previous studies that have examined correlates of e-cigarette use have found that smokers who use e-cigarettes tend to be younger than non-users, smoke more heavily, and are more likely to have tried to quit in the past year (4–6). There is also some evidence that e-cigarette use is more prevalent among people with greater socioeconomic advantage (5,6), although the gap appears to have narrowed over recent years (20). However, there is a distinct lack of evidence on both the prevalence of long-term use and the profile of long-term users. This information is important for the evaluation of the overall public health impact of e-cigarettes, which requires specification of a wide variety of parameters beyond the safety of e-cigarettes and their effect on cessation, including the extent and characteristics of people who become long-term users (21).

The present study therefore aimed to examine the prevalence of, and sociodemographic and smoking-related characteristics associated with, long-term (≥12 months) e-cigarette use in England. We also analysed data on long-term NRT use as a case-control, in order to assess the extent to which the prevalence of long-term e-cigarette use and profile of long-term users are specific to e-cigarettes or apply more broadly to non-combustible nicotine products in general. Specifically, we aimed to answer the following research questions:

1. What proportion of adults in England retrospectively report using (i) e-cigarettes or (ii) NRT for at least one year?
2. What proportion of past-year smokers in England retrospectively report using (i) e-cigarettes or (ii) NRT for at least one year?
3. What proportion of past-year smokers in England report current use of (i) e-cigarettes or (ii) NRT at baseline and both 6 and 12-month follow-ups?
4. How do long-term users of e-cigarettes and NRT differ from non-users in their sociodemographic and smoking-related characteristics?

Method

Design and study population

For peer review only - http://bmjopen.bmj.com/site/about/guidelines.xhtml

Data were used from the Smoking Toolkit Study (STS), an ongoing monthly repeat cross-sectional survey of adults in England (22). Each month, a form of random location sampling is used to select a new sample of approximately 1,700 adults aged 16 years and older. Grouped output areas (containing ~300 households) are stratified by ACORN (socio-demographic) characteristics (http://www.caci.co.uk/acron/acornmap.asp) and region before being randomly selected for inclusion in an interviewers list. Interviewers then choose which houses within these areas are most likely to fulfil their quotas and conduct face-to-face computer-assisted interviews with one member per household. Comparisons of sociodemographic data and smoking prevalence and consumption estimates with national data indicate that STS data are broadly representative of the English population, having a similar composition to other large national surveys, such as the Health Survey for England (22). All participants provide fully informed consent prior to participation. In each wave, respondents complete a face-to-face computer-assisted survey with a trained interviewer. Respondents to the baseline survey between September 2014 and September 2016 who reported smoking in the past year were asked whether they were willing to be re-contacted, and those who agreed were followed up by telephone 6 and 12 months after the baseline interview. Up to 7 attempts were made to follow up each consenting participant. For the purpose of the present study, we aggregated data across survey waves. Cross-sectional analyses used data from all adults who responded to the baseline survey during this period, and the prospective analysis used data from respondents who reported past-year smoking at baseline and responded to both the 6 and 12-month follow-up surveys.

Patient and public involvement

The wider toolkit study has been discussed with a diverse patient and public involvement (PPI) group, and the authors regularly attend and present at meetings at which patients and public are included. Interaction and discussion at these events help to shape the broad research priorities and questions. There is also a mechanism for generalized input from the wider public: each month interviewers seek feedback on the questions from all 1700 respondents, who are representative of the English population. This feedback is limited, and usually simply relates to understanding of questions and item options. No patients or members of the public were involved in setting the research questions or the outcome measures, nor were they involved in the design and implementation of this specific study. There are no plans to involve patients in dissemination.

Measures

Outcomes: long-term use of e-cigarettes and NRT

For peer review only - http://bmjopen.bmj.com/site/about/guidelines.xhtml

The outcomes were long-term (≥12 months) use of e-cigarettes and long-term use of NRT, assessed retrospectively at baseline and prospectively over a 12-month follow-up.

In each of the baseline and follow-up surveys, three questions asked respondents about current use of e-cigarettes (or other vaping devices) and NRT (e.g. nicotine patches, gum, spray, or any other product):

1. Which, if any, of these are you currently using to help you cut down the amount you smoke?
2. Do you regularly use either of these in situations when you are not allowed to smoke?
3. Can I check, do you currently use either of the following at all for any reason?

In the baseline survey, respondents reporting use of either e-cigarettes or NRT were asked: "How long have you been using this nicotine replacement product or these products for?" Response options were: (i) less than one week, (ii) one to six weeks, (iii) more than six weeks and up to 12 weeks, (iv) more than 12 weeks and up to 26 weeks, (v) more than 26 weeks and up to 52 weeks, and (vi) more than 52 weeks.

For the present analyses, long-term use of e-cigarettes/NRT was defined as current use initiated more than 52 weeks prior to the baseline survey for cross-sectional analyses, and as current use at baseline, 6 months and 12 months for prospective analyses. Participants who reported long-term use of both e-cigarettes and NRT (*n*=66) were excluded.

Sociodemographic and smoking-related characteristics

Data were included on a range of sociodemographic and smoking-related characteristics assessed at baseline, selected *a priori* on the basis of previous studies demonstrating associations with use of e-cigarettes and/or NRT.

Sociodemographic information included: age, sex, ethnicity, region, social grade, level of education, disability, and the presence of children in the household. Ethnicity was categorised as white vs. non-white. Region was defined according to Government Office Region, grouped into three categories: northern, central and southern England. Social grade was categorised as ABC1 (which includes managerial, professional and intermediate occupations) vs. C2DE (which includes small employers and own-account workers, lower supervisory and technical occupations, and semi-routine and routine occupations, never workers and long-term unemployed). This occupational measure of social grade is a valid index of SES that is widely used in research in UK populations. It has been identified as particularly relevant in the context of tobacco use and quitting (23) and other addictive behaviours (24). These social grades are frequently

For peer review only - http://bmjopen.bmj.com/site/about/guidelines.xhtml

amalgamated into two groupings; ABC1 and C2DE. Here, researchers frequently interpret ABC1 to represent the middle class and C2DE to represent the working class. Education was categorised as lower (no post-16 qualifications) vs. higher (higher-level qualifications above GCSE level). Disability status was identified from the question "Do you consider yourself to have a disability within the meaning of the Disability Discrimination Act 1995 (yes/no)?". The number of children in the household was self-reported and dichotomised to 0 vs. ≥1.

Smoking-related characteristics included: smoking status, time to first cigarette, consistent motivation to stop and (because it has been shown to be associated with smoking and quitting behaviour (25–27)) high-risk drinking. Smoking status was self-reported by all adults in response to the question: "Which of the following best applies to you (current smoker/stopped in the past year/stopped more than a year ago/never smoked)?". Respondents who reported current smoking or having stopped in the past year ('past-year smokers') were also asked how soon after waking they typically smoked their first cigarette (categorised as within 30 vs. ≥31 minutes; an established indicator of nicotine dependence (28)) and whether they had consistently felt that they wanted to stop in the past year (yes/no). High-risk drinking was assessed using the Alcohol Use Disorders Identification Test (AUDIT) (29), a 10-item screening tool developed by the World Health Organisation to assess alcohol consumption, drinking behaviours and alcohol dependence, with a score of 8 or more indicating high-risk drinking.

Statistical analysis

The analysis plan and syntax were pre-registered on Open Science Framework (https://osf.io/bpjhk/).

We used chi-square tests to compare the baseline characteristics of individuals who responded to both the 6 and 12-month follow-ups with those who were lost to follow-up in order to assess the representativeness of those followed-up.

We estimated the weighted prevalence of long-term use of (i) e-cigarettes and (ii) NRT in the total adult population at baseline, and in past-year smokers at baseline and over 12-month follow-up. Rim (marginal) weighting was used to match the English population on the dimensions of age, social grade, region, housing tenure (bought on a mortgage, owned outright, rented from local authority, rented from private landlord), ethnicity and working status (working, not working) within sex.

For peer review only - http://bmjopen.bmj.com/site/about/guidelines.xhtml

For peer review only

We then used logistic regression to examine the extent to which sociodemographic and smoking-related characteristics were associated with long-term use of e-cigarettes and NRT, assessed at baseline. For each outcome, we analysed bivariate associations with each potential correlate separately, and tested independent associations with a multivariable model that included all variables. We had also intended to analyse associations with long-term use prospectively, but the achieved sample size was lower than we had anticipated, and the prevalence of long-term use was low (particularly for NRT), limiting statistical power.

Following peer review, we added an unplanned sensitivity analysis of the prospective data in which missing data on e-cigarette and NRT use at 6 months and 12 months were imputed for all baseline past-year smokers with missing data. We used a multiple imputation model with all baseline sociodemographic and smoking-related characteristics, baseline use of e-cigarettes, and baseline use of NRT as predictors. Five imputed datasets were created, each analysed separately, and the results combined to produce pooled estimates of prevalence of long-term use of e-cigarettes and long-term use of NRT.

Results

Long-term use of e-cigarettes and NRT among all adults in England: cross-sectional data

A total of 42,040 adults in England were surveyed between September 2014 and September 2016 and 40,933 (97.4%) were complete cases. The weighted prevalence of long-term e-cigarette use assessed retrospectively among all adults in England was 1.5% (95%CI 1.4-1.6%) and of long-term NRT use was 0.5% (95%CI 0.4-0.6%). Table 1 summarises sample characteristics and bivariate and multivariable associations between sociodemographic and smoking-related characteristics and long-term use of e-cigarettes and NRT among all adults in the baseline survey.

In the multivariable model, both long-term e-cigarette use and long-term NRT use were significantly associated with age, region, level of education, disability, and smoking status. Compared with those aged 16-34, long-term e-cigarette use was more prevalent among those aged 35-54 but was not significantly different among those aged ≥55 years. Long-term NRT was significantly more prevalent among those aged 35-54 and ≥55 years. Compared with the north of England, long-term e-cigarette use was less prevalent in central and southern regions, and long-term NRT use was more prevalent in the south. Both long-term e-cigarette use and long-term NRT use were significantly less prevalent among people with no post-16

For peer review only - http://bmjopen.bmj.com/site/about/guidelines.xhtml

qualifications and more prevalent among those with a disability. Prevalence of long-term e-cigarette and NRT use did not differ significantly between current smokers and recent ex-smokers, but was significantly less prevalent among never smokers, among whom use of either product was rarely reported (e-cigarettes 0.1%, NRT 0.0%). Long-term NRT use was also significantly less prevalent among long-term ex-smokers, but the prevalence of long-term e-cigarette use did not differ significantly between long-term ex-smokers and current smokers.

Long-term NRT use, but not long-term e-cigarette use, was associated with sex and high-risk drinking, with higher prevalence observed among women and high-risk drinkers. Long-term e-cigarette use, but not long-term NRT use, was associated with the presence of children in the household, with lower prevalence observed among people with children in their household. We observed no significant association between long-term e-cigarette or NRT use and ethnicity or social grade.

Long-term use of e-cigarettes and NRT among past-year smokers in England: cross-sectional data

A total of 8,649 participants were past-year smokers and 8,406 (97.2%) were complete cases. The weighted prevalence of long-term e-cigarette use assessed retrospectively among past-year smokers in England was 3.9% (95%CI 3.5-4.3%) and of long-term NRT use was 1.3% (95%CI 1.1-1.5%). Table 2 summarises bivariate and multivariable associations between sociodemographic and smoking-related characteristics and long-term use of e-cigarettes and NRT among past-year smokers in the baseline survey.

In the multivariable model, there were significant associations between long-term use of e-cigarettes and NRT and age, region, and motivation to stop smoking, and between long-term use of e-cigarettes and social grade, level of education, and children in the household. Long-term use of e-cigarettes and NRT was more prevalent among older smokers compared with 16-34 year-olds, and among those who were motivated to stop. Compared with the north of England, long-term e-cigarette use was less prevalent in central and southern regions, but long-term NRT use was more prevalent in the south. Long-term e-cigarette use was significantly less prevalent among smokers from social grades C2DE, without post-16 qualifications and with children in their household, while long-term NRT use did not differ significantly according to these factors. We observed no significant association between long-term e-cigarette or NRT use and sex, ethnicity, disability, current smoking status, excessive drinking, or dependence.

Long-term use of e-cigarettes and NRT among all adults in England: prospective data

For peer review only - http://bmjopen.bmj.com/site/about/guidelines.xhtml

A total of 733 individuals who reported past-year smoking at baseline completed follow-up surveys at both 6 and 12 months. Characteristics of past-year smokers in the baseline and follow-up samples are summarised in Table 3. Past-year smokers who responded to follow-up were significantly older than those who did not. A higher proportion of responders were white and fewer were from social grades CD2E or had no post-16 qualifications. More reported a disability and fewer had children in the household. A higher proportion of responders than non-responders were recent ex-smokers and more reported consistent motivation to stop smoking. They were also significantly more likely to report long-term use of e-cigarettes or NRT than those who did not respond to the follow-up surveys. Loss to follow-up was not significantly associated with sex, region, high-risk drinking, or dependence. The weighted prevalence of long-term e-cigarette use assessed prospectively among past-year smokers in England was 13.4% (95%CI 10.9-15.9%) and of long-term NRT use was 1.9% (95%CI 0.9-2.9%).

When missing data on use of e-cigarettes and NRT at 6 and 12 months were multiply imputed for participants who were past-year smokers at baseline and did not participate in the follow-up surveys (*n*=1,673, 69.5% of all baseline past-year smokers), the unweighted prevalence of long-term e-cigarette use assessed prospectively was 9.8% (95%CI 9.2-10.4%) and of long-term NRT use was 1.7% (95%CI 1.4-2.0%), and the weighted prevalence was 10.3% (95%CI 9.7-11.0%) and 1.6% (95%CI 1.3-1.9%), respectively.

Discussion

In this large, representative sample of adults in England, long-term use of e-cigarettes and NRT was almost exclusively reported by current or ex-smokers. Only a minority of past-year smokers retrospectively reported long-term use of either e-cigarettes (3.9%) or NRT (1.3%) but this figure may be an underestimate: prevalence of current use at three time-points over a 12-month period was substantially higher for both e-cigarettes (13.4%) and NRT (1.9%), although these estimates were likely subject to attrition bias. When missing data were imputed, prospectively assessed prevalence estimates were slightly lower, at 10.3% for long-term e-cigarette use and 1.6% for long-term NRT use. Both cross-sectionally and prospectively, there was a higher prevalence of long-term e-cigarette use in comparison with NRT use. In adjusted models, long-term use of e-cigarettes and NRT was higher among older smokers and those more motivated to quit smoking. Long-term use of e-cigarettes was less common, and long-term use of NRT was more common, in the south of England compared with the north. Long-term use of e-cigarettes was significantly less prevalent

For peer review only - http://bmjopen.bmj.com/site/about/guidelines.xhtml

among smokers who were less educated, those from social grades C2DE, and those with children in the household, but these variables were not significantly associated with long-term use of NRT. Neither long-term use of e-cigarettes nor NRT differed significantly according to sex, ethnicity, disability, current smoking status (current vs. recent ex-smokers), high-risk drinking, or nicotine dependence.

To our knowledge, this study is the first to examine the prevalence of, and sociodemographic and smoking-related characteristics associated with, long-term use of e-cigarettes. We aimed to identify the prevalence of long-term e-cigarette use cross-sectionally and prospectively, and to contrast usage with long-term NRT use. Our results showed a higher prevalence of long-term e-cigarette use in comparison with NRT. Recent prevalence estimates indicate that current use of e-cigarettes is much more popular than NRT (7) and the same appears true for long-term use.

Long-term use of both products was almost exclusively observed among current and former smokers. Concerns have been raised that e-cigarettes may serve as a gateway to cigarette smoking among never-smokers, particularly among youth (30,31), but in our sample, the prevalence of long-term e-cigarette use among never-smokers was just 0.1%, comparable to long-term NRT use. As such, the potential number of people susceptible to any gateway effects in England between 2014 and 2016 appears to have been very small. We also observed higher prevalence of long-term e-cigarette use among middle-aged and older adults than in the youngest group (16-34 years), in contrast with evidence that current use of e-cigarettes among current and former smokers in England is least prevalent in the oldest age group (12.2% in those aged ≥65 years, compared with 18.7%, 21.4%, 20.8%, 20.6%, and 18.6% in those aged 16-24, 25-34, 35-44, 45-54, and 55-64 years) (7).

While the prevalence of long-term use of e-cigarettes and NRT did not differ significantly between current and recent (<1 year) ex-smokers, the relative prevalence of use in long-term (≥1 year) ex-smokers differed between the products. Long-term use of NRT was significantly less prevalent among long-term ex-smokers than current smokers, whereas long-term use of e-cigarettes was similar between these groups. This suggests that people tend to discontinue use of NRT more quickly after quitting smoking than with e-cigarettes, possibly because e-cigarettes are a closer substitute for the behaviour of cigarette smoking than NRT, or because NRT is viewed more as a medication than a recreational product (9). A recent trial of e-cigarettes compared with NRT in UK stop smoking services observed similar, with participants randomised to use e-cigarettes in a quit attempt more likely than those randomised to use NRT to still be using their allocated product one year later (80% vs. 9%, respectively) (9).

For peer review only - http://bmjopen.bmj.com/site/about/guidelines.xhtml

Insofar that use of alternative nicotine products should promote cessation rather than continued dual use, it is somewhat concerning that long-term use of e-cigarettes was similarly prevalent among current and recent ex-smokers. On the other hand, this appeared equally true for NRT. There have been concerns that dual use of cigarettes and e-cigarettes could reduce the urgency to quit smoking (32) and extend the duration of cigarette smoking (33,34). This would result in a negative overall public health impact, since duration of smoking poses a greater health risk than intensity of smoking (35). However, our results indicate that this is not the case: after mutual adjustment, the recall of long-term use of both e-cigarettes and NRT was higher among smokers who were more motivated to quit. This finding is consistent with previous studies showing that the most common reason for using an e-cigarette is to stop smoking (3), that smokers who use e-cigarettes are more likely to have recently tried to quit (4–6), and that long-term e-cigarette use is associated with a higher rate of smoking cessation (14). It suggests that long-term dual use is not associated with reduced motivation to quit.

Among past-year smokers, long-term use of e-cigarettes specifically was lower among those without post-16 qualifications and those from social grades C2DE. This is consistent with a larger literature on the diffusion of innovation, which recognises the tendency for high status groups to most quickly adopt new ideas and behaviours (36–38); a pattern that was documented for combustible cigarette smoking (39). According to this theory, one would expect to see e-cigarette use spread first within more affluent social networks, but patterns of imitation later lead to diffusion of the practice and normative change across the socio-economic range. The fact that we observed no significant association between education or social grade and long-term NRT use, which has a similar cost to users (40) but has been available for much longer, is consistent with this. With recent evidence indicating that the socioeconomic gradient in e-cigarette use is declining over time (20), we predict that this disparity in long-term use will disappear over the coming years. There were also regional differences, with long-term e-cigarette use more prevalent in the north of England and long-term NRT use more prevalent in the south. The higher prevalence of long-term e-cigarette use in the north is consistent with previous evidence of heavier smoking in the north of England (41) and higher prevalence of e-cigarette use among heavier smokers (4–6).

Prospective analysis of current use at baseline and both 6 and 12-month follow-ups indicated a substantially higher rate of long-term use of both e-cigarettes (13.4%) and NRT (1.9%) among past-year smokers than was seen in the cross-sectional results. While these figures are not directly comparable given the substantial attrition over follow-up assessments and differences in the definition of long-term use (continued use vs. current use at three defined time points), the magnitude of the difference indicates that retrospective recall

For peer review only - http://bmjopen.bmj.com/site/about/guidelines.xhtml

of how long the products have been used may underestimate what could be expected if users were followed more frequently over time. A study with a greater number of follow-up points over a longer period could offer further insight into this discrepancy.

Strengths of this study include the large, representative sample and prospective design. However, there were several limitations. Only respondents who reported past-year smoking at baseline were invited to participate in the follow-up surveys, so we were unable to obtain prospective estimates of the prevalence of long-term e-cigarette or NRT use in the entire adult population. While, evidence from the cross-sectional results of this study and from previous research (3) suggest that the vast majority of long-term users were current or recent ex-smokers, with low prevalence among never smokers, it would have been useful to have data from long-term ex-smokers. Another potential issue was substantial attrition bias. Our sample for prospective analyses was older and more socioeconomically advantaged than the group who were lost to follow-up, and more reported long-term use of e-cigarettes or NRT retrospectively at baseline. They were also more likely to have quit recently. This may have contributed to the higher prevalence of long-term use observed in prospective analyses. Finally, we did not consider reasons for or patterns of use. Future studies could build on our findings through more detailed or frequent assessments, and qualitative work with long-term users.

Conclusions

Long-term e-cigarette use is more prevalent than long-term NRT use in the English adult population, but both are almost exclusively by current or ex-smokers. The profile of long-term e-cigarette users is broadly similar to that of long-term NRT users, although there are some sociodemographic and regional differences between the two, with long-term e-cigarette use more prevalent among smokers with greater socioeconomic advantage and in the north of England, and long-term NRT use more prevalent among smokers in the south. Prospective assessment of long-term use produces substantially higher estimates of prevalence, particularly for e-cigarettes, than retrospective recall, although this may to some extent be accounted for by differences in the sample and definitions used. These results add to the descriptive picture of e-cigarette use in England, providing novel insight into long-term use. This information can be incorporated into broader evaluations of population-level use of e-cigarettes and their potential impact on public health.

For peer review only - http://bmjopen.bmj.com/site/about/guidelines.xhtml

References

1. World Health Organization. Tobacco [Internet]. 2018 [cited 2018 Dec 2]. Available from: http://www.who.int/news-room/fact-sheets/detail/tobacco

2. Royal College of Physicians. Harm reduction in nicotine addiction: helping people who can't quit [Internet]. 2007 [cited 2018 Dec 5]. Available from: https://shop.rcplondon.ac.uk/products/harm-reduction-in-nicotine-addiction-helping-people-who-cant-quit

3. McNeill A, Brose LS, Calder R, Bauld L, Robson D. Evidence review of e-cigarettes and heated tobacco products 2018. A report commissioned by Public Health England. [Internet]. 2018 [cited 2018 Jun 21]. Available from: https://www.gov.uk/government/publications/e-cigarettes-and-heated-tobacco-products-evidence-review/evidence-review-of-e-cigarettes-and-heated-tobacco-products-2018-executive-summary

4. Vardavas CI, Filippidis FT, Agaku IT. Determinants and prevalence of e-cigarette use throughout the European Union: a secondary analysis of 26 566 youth and adults from 27 Countries. Tob Control. 2015 Sep 1;24(5):442–8.

5. Levy DT, Yuan Z, Li Y. The Prevalence and Characteristics of E-Cigarette Users in the U.S. Int J Environ Res Public Health [Internet]. 2017 Oct [cited 2018 Dec 5];14(10). Available from: https://www.ncbi.nlm.nih.gov/pmc/articles/PMC5664701/

6. Brown J, West R, Beard E, Michie S, Shahab L, McNeill A. Prevalence and characteristics of e-cigarette users in Great Britain: Findings from a general population survey of smokers. Addict Behav. 2014 Jun 1;39(6):1120–5.

7. West R, Proudfoot H, Beard E, Brown J. Electronic cigarettes in England - latest trends [Internet]. Smoking in England; 2019 [cited 2019 May 26]. Available from: http://www.smokinginengland.info/latest-statistics/

8. McRobbie H, Bullen C, Hartmann-Boyce J, Hajek P. Electronic cigarettes for smoking cessation and reduction. Cochrane Database Syst Rev. 2014;(12):CD010216.

9. Hajek P, Phillips-Waller A, Przulj D, Pesola F, Myers Smith K, Bisal N, et al. A Randomized Trial of E-Cigarettes versus Nicotine-Replacement Therapy. N Engl J Med. 2019 Feb 14;380(7):629–37.

10. Beard E, West R, Michie S, Brown J. Association between electronic cigarette use and changes in quit attempts, success of quit attempts, use of smoking cessation pharmacotherapy, and use of stop smoking services in England: time series analysis of population trends. BMJ. 2016 Sep 13;354:i4645.

11. Zhu S-H, Zhuang Y-L, Wong S, Cummins SE, Tedeschi GJ. E-cigarette use and associated changes in population smoking cessation: evidence from US current population surveys. BMJ. 2017 Jul 26;358:j3262.

12. Royal College of Physicians. Nicotine without smoke: Tobacco harm reduction [Internet]. 2016 Apr [cited 2018 Dec 5]. Available from: https://www.rcplondon.ac.uk/projects/outputs/nicotine-without-smoke-tobacco-harm-reduction-0

For peer review only - http://bmjopen.bmj.com/site/about/guidelines.xhtml

13. Hughes JR, Keely J, Naud S. Shape of the relapse curve and long-term abstinence among untreated smokers. Addict Abingdon Engl. 2004 Jan;99(1):29–38.

14. Zhuang Y-L, Cummins SE, Sun JY, Zhu S-H. Long-term e-cigarette use and smoking cessation: a longitudinal study with US population. Tob Control. 2016 Oct 1;25(Suppl 1):i90–5.

15. Goniewicz ML, Gawron M, Smith DM, Peng M, Jacob P, Benowitz NL. Exposure to Nicotine and Selected Toxicants in Cigarette Smokers Who Switched to Electronic Cigarettes: A Longitudinal Within-Subjects Observational Study. Nicotine Tob Res. 2017 Feb 1;19(2):160–7.

16. McRobbie H, Phillips A, Goniewicz ML, Smith KM, Knight-West O, Przulj D, et al. Effects of Switching to Electronic Cigarettes with and without Concurrent Smoking on Exposure to Nicotine, Carbon Monoxide, and Acrolein. Cancer Prev Res Phila Pa. 2015 Sep;8(9):873–8.

17. Hecht SS, Carmella SG, Kotandeniya D, Pillsbury ME, Chen M, Ransom BWS, et al. Evaluation of toxicant and carcinogen metabolites in the urine of e-cigarette users versus cigarette smokers. Nicotine Tob Res Off J Soc Res Nicotine Tob. 2015 Jun;17(6):704–9.

18. Shahab L, Goniewicz ML, Blount BC, Brown J, McNeill A, Alwis KU, et al. Nicotine, Carcinogen, and Toxin Exposure in Long-Term E-Cigarette and Nicotine Replacement Therapy Users: A Cross-sectional Study. Ann Intern Med. 2017 Mar 21;166(6):390–400.

19. Wilson S, Partos T, McNeill A, Brose LS. Harm perceptions of e-cigarettes and other nicotine products in a UK sample. Addict Abingdon Engl. 2019 Jan 3;

20. Kock L, Shahab L, West R, Brown J. E-cigarette use in England 2014-17 as a function of socio-economic profile. Addict Abingdon Engl. 2018 Oct 10;

21. Levy DT, Cummings KM, Villanti AC, Niaura R, Abrams DB, Fong GT, et al. A framework for evaluating the public health impact of e-cigarettes and other vaporized nicotine products. Addict Abingdon Engl. 2017;112(1):8–17.

22. Fidler JA, Shahab L, West O, Jarvis MJ, McEwen A, Stapleton JA, et al. “The smoking toolkit study”: a national study of smoking and smoking cessation in England. BMC Public Health. 2011 Jun 18;11:479.

23. Kotz D, West R. Explaining the social gradient in smoking cessation: it’s not in the trying, but in the succeeding. Tob Control. 2009 Feb;18(1):43–6.

24. Beard E, Brown J, West R, Kaner E, Meier P, Michie S. Associations between socio-economic factors and alcohol consumption: A population survey of adults in England. PLOS ONE. 2019 Feb 4;14(2):e0209442.

25. Falk DE, Yi H, Hiller-Sturmhöfel S. An epidemiologic analysis of co-occurring alcohol and tobacco use and disorders: findings from the National Epidemiologic Survey on Alcohol and Related Conditions. Alcohol Res Health J Natl Inst Alcohol Abuse Alcohol. 2006;29(3):162–71.

26. Beard E, West R, Michie S, Brown J. Association between smoking and alcohol-related behaviours: a time–series analysis of population trends in England. Addict Abingdon Engl. 2017 Oct;112(10):1832–41.

For peer review only

27. Brown J, West R, Beard E, Brennan A, Drummond C, Gillespie D, et al. Are recent attempts to quit smoking associated with reduced drinking in England? A cross-sectional population survey. BMC Public Health. 2016 Jul 22;16(1):535.

28. Heatherton TF, Kozlowski LT, Frecker RC, FAGERSTROM K-O. The Fagerström test for nicotine dependence: a revision of the Fagerstrom Tolerance Questionnaire. Br J Addict. 1991;86(9):1119–1127.

29. Babor TF, Higgins-Biddle JC, Saunders JB, Monteiro MG, Organization WH. AUDIT: the alcohol use disorders identification test: guidelines for use in primary health care. 2001;

30. Soneji S, Barrington-Trimis JL, Wills TA, Leventhal AM, Unger JB, Gibson LA, et al. Association Between Initial Use of e-Cigarettes and Subsequent Cigarette Smoking Among Adolescents and Young Adults: A Systematic Review and Meta-analysis. JAMA Pediatr. 2017 Aug 1;171(8):788–97.

31. Schneider S, Diehl K. Vaping as a Catalyst for Smoking? An Initial Model on the Initiation of Electronic Cigarette Use and the Transition to Tobacco Smoking Among Adolescents. Nicotine Tob Res Off J Soc Res Nicotine Tob. 2016 May;18(5):647–53.

32. Grana R, Benowitz N, Glantz SA. E-cigarettes: a scientific review. Circulation. 2014 May 13;129(19):1972–86.

33. Furlow B. US Government warns against long-term dual use of conventional and e-cigarettes. Lancet Respir Med. 2015 May;3(5):345.

34. Maziak W. Harm reduction at the crossroads: the case of e-cigarettes. Am J Prev Med. 2014 Oct;47(4):505–7.

35. Doll R, Peto R. Cigarette smoking and bronchial carcinoma: dose and time relationships among regular smokers and lifelong non-smokers. J Epidemiol Community Health. 1978 Dec 1;32(4):303–13.

36. Strang D, Soule SA. Diffusion in Organizations and Social Movements: From Hybrid Corn to Poison Pills. Annu Rev Sociol. 1998;24(1):265–90.

37. Rogers EM. Diffusion of Innovations, 4th Edition. Simon and Schuster; 2010. 550 p.

38. Katz E. Theorizing Diffusion: Tarde and Sorokin Revisited. Ann Am Acad Pol Soc Sci. 1999 Nov 1;566(1):144–55.

39. Ferrence R. Deadly Fashion: The Rise and Fall of Cigarette Smoking in North America. 1 edition. New York: Garland Science; 1990. 167 p.

40. Jackson SE, Shahab L, Kock L, West R, Brown J. Expenditure on smoking and alternative nicotine delivery products: a population survey in England. Addiction. 2019;

41. Beard E, Brown J, West R, Angus C, Kaner E, Michie S. Healthier central England or North–South divide? Analysis of national survey data on smoking and high-risk drinking. BMJ Open. 2017 Mar 1;7(3):e014210.

For peer review only

For peer review only - http://bmjopen.bmj.com/site/about/guidelines.xhtml

For peer review only

For peer review only - http://bmjopen.bmj.com/site/about/guidelines.xhtml

Declarations

Ethics approval and consent to participate

Ethical approval for the Smoking Toolkit Study was granted originally by the UCL Ethics Committee (ID 0498/001) and participants provided full informed consent. The data are not collected by UCL and are anonymised when received by UCL.

Availability of data and materials

The datasets used and/or analysed during the current study are available from the corresponding author on reasonable request.

Competing interests

JB received unrestricted research funding from Pfizer, who manufacture smoking cessation medications. LS has received honoraria for talks, an unrestricted research grant and travel expenses to attend meetings and workshops from Pfizer, and has acted as paid reviewer for grant awarding bodies and as a paid consultant for health care companies. All authors declare no financial links with tobacco companies or e-cigarette manufacturers or their representatives.

Funding

This work was supported by Cancer Research UK, grant numbers C1417/A22962 and C44576/A19501. The funders had no final role in the study design; in the collection, analysis and interpretation of data; in the writing of the report; or in the decision to submit the paper for publication. All researchers listed as authors are independent from the funders and all final decisions about the research were taken by the investigators and were unrestricted.

Author contributions

JB and EH conceived of the study. SEJ, EH and JB analysed and interpreted the data. SEJ and EH drafted the manuscript. LS, EB, SM and JB critically revised the manuscript for important intellectual content.

For peer review only - http://bmjopen.bmj.com/site/about/guidelines.xhtml

Table 1 Sample descriptive characteristics and factors associated with long-term use of e-cigarettes or NRT by adults in England: cross-sectional da (*n*=40,933)

	Whole sample	E-cigarettes			NRT		
	**% (*n*)**	**% (*n*) long-term use**	**OR [95% CI] *p***	**Adj OR [95% CI] *p***	**% (*n*) long-term use**	**OR [95% CI] *p***	**Adj O**
All adults*	-	1.5 (604)	-	-	0.5 (205)	-	
Age in years							
16-34	30.3 (12,398)	1.1 (138)	1.00	1.00	0.2 (21)	1.00	
35-54	29.6 (12,118)	2.1 (252)	1.89 (1.53:2.33) <0.001	1.71 (1.38:2.13) <0.001	0.7 (87)	4.26 (2.65:6.87) <0.001	3.98
≥55	40.1 (16,417)	1.1 (185)	1.01 (0.81:1.26) 0.912	0.78 (0.60:1.02) 0.064	0.6 (89)	3.21 (2.00:5.17) <0.001	3.25
Sex							
Men	50.9 (20.816)	1.5 (308)	1.00	1.00	0.5 (94)	1.00	
Women	49.1 (20.117)	1.3 (267)	0.90 (0.76:1.06) 0.190	1.04 (0.87:1.23) 0.683	0.5 (103)	1.14 (0.86:1.50) 0.377	1.39
Ethnicity							
Non-white	16.4 (6,730)	0.6 (42)	1.00	1.00	0.1 (10)	1.00	
White	83.6 (34,203)	1.6 (533)	2.52 (1.84:3.46) <0.001	1.12 (0.80:1.56) 0.510	0.5 (187)	3.69 (1.95:6.98) <0.001	1.48
Social grade							
ABC1	53.5 (21,894)	1.3 (292)	1.00	1.00	0.5 (102)	1.00	
C2DE	46.5 (19,039)	1.5 (283)	1.12 (0.95:1.32) 0.191	0.91 (0.76:1.10) 0.326	0.5 (95)	1.07 (0.81:1.42) 0.629	0.93
Region							
North	32.0 (13,111)	2.1 (281)	1.00	1.00	0.4 (57)	1.00	
Central	29.3 (12,000)	1.1 (132)	0.51 (0.41:0.63) <0.001	0.60 (0.48:0.74) <0.001	0.5 (59)	1.13 (0.79:1.63) 0.507	1.41
South	38.7 (15,822)	1.0 (162)	0.47 (0.39:0.57) <0.001	0.55 (0.45:0.67) <0.001	0.5 (81)	1.18 (0.84:1.66) 0.343	1.45
Level of education							
Post-16 qualifications	63.4 (25,945)	1.4 (371)	1.00	1.00	0.5 (126)	1.00	
No post-16 qualifications	36.6 (14,988)	1.4 (204)	0.95 (0.80:1.13) 0.569	0.79 (0.65:0.95) 0.012	0.5 (71)	0.98 (0.73:1.31) 0.867	0.71
Disability							
No	88.2 (36,119)	1.3 (463)	1.00	1.00	0.4 (152)	1.00	
Yes	11.8 (4,814)	2.3 (112)	1.83 (1.49:2.26) <0.001	1.34 (1.07:1.66) 0.009	0.9 (45)	2.23 (1.60:3.12) <0.001	1.52
Children in the household							
0	71.0 (29,062)	1.4 (413)	1.00	1.00	0.5 (145)	1.00	

For peer review only - http://bmjopen.bmj.com/site/about/guidelines.xhtml

≥1	29.0 (11,871)	1.4 (162)	0.96 (0.80:1.15) 0.660	0.80 (0.65:0.98) 0.030	0.4 (52)	0.88 (0.64:1.21) 0.420	1.00
Current smoking status							
Current smoker	19.3 (7,900)	3.8 (302)	1.00	1.00	1.4 (107)	1.00	
Recent (<1y) ex-smoker	1.4 (558)	3.8 (21)	0.98 (0.63:1.54) 0.944	0.93 (0.59:1.46) 0.747	1.3 (7)	0.93 (0.43:2.00) 0.843	0.87
Long-term (≥1y) ex-smoker	17.1 (7,009)	3.2 (225)	0.83 (0.70:1.00) 0.043	0.90 (0.74:1.09) 0.274	1.1 (76)	0.80 (0.59:1.07) 0.136	0.62
Never smoker	62.2 (25,466)	0.1 (27)	0.03 (0.02:0.04) <0.001	0.03 (0.02:0.04) <0.001	0.0 (7)	0.02 (0.01:0.04) <0.001	0.02
High-risk drinking							
No	87.3 (35,742)	1.3 (449)	1.00	1.00	0.4 (149)	1.00	
Yes	12,7 (5,191)	2.4 (126)	1.95 (1.60:2.39) <0.001	1.02 (0.83:1.27) 0.830	0.9 (48)	2.22 (1.61:3.09) <0.001	1.60

The adjusted model includes all variables in the table and year of survey.

*This figure is weighted, and therefore the effective N does not correspond precisely with the unweighted figures reported elsewhere in the table.

OR = odds ratio; CI = confidence interval; SD = standard deviation; NRT = nicotine replacement therapy.

For peer review only

For peer review only - http://bmjopen.bmj.com/site/about/guidelines.xhtml

Table 2 Factors associated with long-term use of e-cigarettes or NRT by past-year smokers in England: cross-sectional data (n=8,406)

	E-cigarettes			NRT		
	**% (*n*) long-term use**	**OR [95% CI]**<br>***p***	**Adj OR [95% CI]**<br>***p***	**% (*n*) long-term use**	**OR [95% CI]**<br>***p***	**Adj OR [95% CI]**<br>***p***
All adults*	3.9 (327)	-	-	1.3 (112)	-	-
Age in years						
16-34	2.6 (88)	1.00	1.00	0.5 (15)	1.00	1.00
35-54	4.9 (135)	1.88 (1.43:2.47)<br><0.001	1.90 (1.44:2.52)<br><0.001	1.7 (46)	3.72 (2.07:6.67)<br><0.001	3.47 (1.92:6.27)<br><0.001
≥55	4.3 (98)	1.64 (1.22:2.19)<br>0.001	1.55 (1.12:2.13)<br>0.008	2.3 (52)	5.10 (2.86:9.08)<br><0.001	5.21 (2.79:9.72)<br><0.001
Sex						
Men	1.3 (57)	1.00	1.00	1.3 (57)	1.00	1.00
Women	1.5 (56)	0.96 (0.77:1.20)<br>0.702	1.00 (0.80:1.27)<br>0.974	1.4 (56)	1.15 (0.79:1.66)<br>0.474	1.14 (0.78:1.68)<br>0.498
Ethnicity						
Non-white	3.4 (31)	1.00	1.00	0.9 (8)	1.00	1.00
White	3.9 (290)	1.15 (0.79:1.68)<br>0.460	1.01 (0.68:1.50)<br>0.961	1.4 (105)	1.62 (0.79:3.33)<br>0.192	1.33 (0.63:2.80)<br>0.459
Social grade						
ABC1	5.2 (167)	1.00	1.00		1.00	1.00
C2DE	3.0 (154)	0.56 (0.45:0.70)<br><0.001	0.66 (0.52:0.84)<br>0.001	1.1 (58)	0.65 (0.45:0.94)<br>0.022	0.71 (0.47:1.06)<br>0.091
Region						
North	4.7 (147)	1.00	1.00	1.0 (32)	1.00	1.00
Central	3.4 (81)	0.73 (0.55:0.96)<br>0.023	0.79 (0.59:1.04)<br>0.094	1.4 (33)	1.38 (0.85:2.25)<br>0.197	1.52 (0.92:2.50)<br>0.100
South	3.2 (93)	0.68 (0.52:0.88)<br>0.004	0.68 (0.52:0.90)<br>0.006	1.7 (48)	1.64 (1.05:2.58)<br>0.030	1.76 (1.11:2.79)<br>0.016
Level of education						
Post-16 qualifications	4.7 (214)	1.00	1.00	1.4 (65)	1.00	1.00
No post-16 qualifications	2.7 (107)	0.57 (0.45:0.72)<br><0.001	0.63 (0.49:0.81)<br><0.001	1.2 (48)	0.85 (0.59:1.24)<br>0.411	0.85 (0.56:1.27)<br>0.426
Disability						
No	3.7 (260)	1.00	1.00	1.2 (86)	1.00	1.00
Yes	4.7 (61)	1.29 (0.97:1.72)<br>0.079	1.22 (0.90:1.64)<br>0.197	2.1 (27)	1.73 (1.12:2.67)<br>0.014	1.46 (0.93:2.29)<br>0.103
Children in the household						
0	4.3 (240)	1.00	1.00	1.5 (83)	1.00	1.00
≥1	2.9 (81)	0.66 (0.51:0.85)<br>0.001	0.68 (0.51:0.90)<br>0.007	1.1 (30)	0.71 (0.47:1.08)<br>0.111	1.07 (0.66:1.72)<br>0.795
Current smoking status						
Current smoker	3.8 (301)	1.00	1.00	1.3 (106)	1.00	1.00
Recent (<1y) ex-smoker	3.7 (20)	0.96 (0.61:1.52)<br>0.858	0.69 (0.43:1.11)<br>0.124	1.3 (7)	0.95 (0.44:2.06)<br>0.904	0.69 (0.32:1.52)<br>0.361
High-risk drinking						

For peer review only

No	3.7 (242)	1.00	1.00	1.3 (84)	1.00	1.00
Yes	4.2 (79)	1.14 (0.88:1.48) 0.323	1.02 (0.78:1.35) 0.871	1.5 (29)	1.20 (0.79:1.84) 0.396	1.52 (0.97:2.38) 0.065
Time to first cigarette						
31 or more minutes	3.8 (163)	1.00	1.00	1.2 (53)	1.00	1.00
Within 30 minutes	3.9 (158)	1.02 (0.82:1.28) 0.833	1.07 (0.85:1.35) 0.581	1.5 (60)	1.20 (0.83:1.74) 0.341	1.16 (0.79:1.70) 0.447
Consistent motivation to stop						
No	2.6 (123)	1.00	1.00	0.9 (41)	1.00	1.00
Yes	5.4 (198)	2.11 (1.68:2.66) <0.001	2.05 (1.63:2.60) <0.001	1.9 (72)	2.27 (1.54:3.33) <0.001	2.33 (1.57:3.46) <0.001

The adjusted model includes all variables in the table and year of survey.

*This figure is weighted, and therefore the effective N does not correspond precisely with the unweighted figures reported elsewhere in the table.

OR = odds ratio; CI = confidence interval; SD = standard deviation; NRT = nicotine replacement therapy.

For peer review only

Table 3 Comparison of the baseline and follow-up samples of past-year smokers

	**Baseline sample*** **% (*n*=8,406)**	**Follow-up sample** **% (*n*=733)**	***p*******
Age in years			
16-34	39.6 (3326)	15.6 (114)	<0.001
35-54	33.0 (2777)	34.4 (252)	-
≥55	27.4 (2303)	50.1 (367)	-
Women	46.2 (3885)	44.9 (329)	0.449
White ethnicity	89.1 (7488)	95.4 (699)	<0.001
Social grade C2DE	61.8 (5193)	51.3 (376)	<0.001
Region			
North	37.5 (3150)	39.4 (289)	0.137
Central	28.1 (2363)	25.0 (183)	-
South	34.4 (2893)	35.6 (261)	-
No post-16 qualifications	46.3 (3893)	40.4 (296)	0.001
Has a disability	15.5 (1304)	22.6 (166)	<0.001
≥1 children in the household	33.6 (2826)	22.9 (168)	<0.001
Current smoking status			
Current smoker	93.5 (7862)	91.7 (672)	0.033
Recent (<1y) ex-smoker	6.5 (544)	8.3 (61)	-
High-risk drinking	22.4 (1879)	20.1 (147)	0.118
First cigarette within 30 minutes	48.6 (4089)	50.3 (369)	0.336
Consistent motivation to stop	43.9 (3694)	48.6 (356)	0.008
Long-term e-cigarette use***	3.8 (321)	5.5 (40)	0.015
Long-term NRT use***	1.3 (113)	2.6 (19)	0.002

*Past-year smokers only.

**Comparison of respondents who did and did not provide follow-up data.

***Assessed at baseline.

For peer review only

STROBE Statement—Checklist of items that should be included in reports of ***cross-sectional studies***

	Item No	Recommendation	Page
**Title and abstract**	1	(*a*) Indicate the study's design with a commonly used term in the title or the abstract	1
		(*b*) Provide in the abstract an informative and balanced summary of what was done and what was found	2
**Introduction**			
Background/rationale	2	Explain the scientific background and rationale for the investigation being reported	4
Objectives	3	State specific objectives, including any prespecified hypotheses	5
**Methods**			
Study design	4	Present key elements of study design early in the paper	5
Setting	5	Describe the setting, locations, and relevant dates, including periods of recruitment, exposure, follow-up, and data collection	6
Participants	6	(*a*) Give the eligibility criteria, and the sources and methods of selection of participants	6
Variables	7	Clearly define all outcomes, exposures, predictors, potential confounders, and effect modifiers. Give diagnostic criteria, if applicable	6/7
Data sources/ measurement	8*	For each variable of interest, give sources of data and details of methods of assessment (measurement). Describe comparability of assessment methods if there is more than one group	6
Bias	9	Describe any efforts to address potential sources of bias	7
Study size	10	Explain how the study size was arrived at	8
Quantitative variables	11	Explain how quantitative variables were handled in the analyses. If applicable, describe which groupings were chosen and why	6/7
Statistical methods	12	(*a*) Describe all statistical methods, including those used to control for confounding	7/8
		(*b*) Describe any methods used to examine subgroups and interactions	7/8
		(*c*) Explain how missing data were addressed	8
		(*d*) If applicable, describe analytical methods taking account of sampling strategy	7/8
		(*e*) Describe any sensitivity analyses	na
**Results**			
Participants	13*	(a) Report numbers of individuals at each stage of study—eg numbers potentially eligible, examined for eligibility, confirmed eligible, included in the study, completing follow-up, and analysed	8
		(b) Give reasons for non-participation at each stage	8
		(c) Consider use of a flow diagram	na
Descriptive data	14*	(a) Give characteristics of study participants (eg demographic, clinical, social) and information on exposures and potential confounders	8/9
		(b) Indicate number of participants with missing data for each variable of interest	na
Outcome data	15*	Report numbers of outcome events or summary measures	Tables
Main results	16	(*a*) Give unadjusted estimates and, if applicable, confounder-adjusted estimates and their precision (eg, 95% confidence interval). Make clear which confounders were adjusted for and why they were included	Tables

For peer review only - http://bmjopen.bmj.com/site/about/guidelines.xhtml

		(*b*) Report category boundaries when continuous variables were categorized	8
		(*c*) If relevant, consider translating estimates of relative risk into absolute risk for a meaningful time period	na
Other analyses	17	Report other analyses done—eg analyses of subgroups and interactions, and sensitivity analyses	na
**Discussion**			
Key results	18	Summarise key results with reference to study objectives	10
Limitations	19	Discuss limitations of the study, taking into account sources of potential bias or imprecision. Discuss both direction and magnitude of any potential bias	12
Interpretation	20	Give a cautious overall interpretation of results considering objectives, limitations, multiplicity of analyses, results from similar studies, and other relevant evidence	13
Generalisability	21	Discuss the generalisability (external validity) of the study results	12
**Other information**			
Funding	22	Give the source of funding and the role of the funders for the present study and, if applicable, for the original study on which the present article is based	17

*Give information separately for exposed and unexposed groups.

Note: An Explanation and Elaboration article discusses each checklist item and gives methodological background and published examples of transparent reporting. The STROBE checklist is best used in conjunction with this article (freely available on the Web sites of PLoS Medicine at http://www.plosmedicine.org/, Annals of Internal Medicine at http://www.annals.org/, and Epidemiology at http://www.epidem.com/). Information on the STROBE Initiative is available at www.strobe-statement.org.

For peer review only

For peer review only - http://bmjopen.bmj.com/site/about/guidelines.xhtml

Prevalence and correlates of long-term e-cigarette and nicotine replacement therapy use: a prospective study in England

Journal:	*BMJ Open*
Manuscript ID	bmjopen-2019-029252.R2
Article Type:	Original research
Date Submitted by the Author:	29-Aug-2019
Complete List of Authors:	Jackson, Sarah ; University College London, Epidemiology and Public Health;<br>Hill, Emily; University College London, Epidemiology and Public Health<br>Shahab, Lion; UCL, Dept of Epidemiology and Public Health<br>Beard, Emma; UCL,<br>Michie, Susan; University College London, Centre for Outcomes Research and Effectivenes<br>Brown, Jamie; University College London, Psychology & Language Sciences
**Primary Subject Heading**:	Smoking and tobacco
Secondary Subject Heading:	Addiction, Public health
Keywords:	PUBLIC HEALTH, E-cigarettes, Nicotine replacement therapy, Population-based survey, Long-term use, Smoking cessation

Prevalence and correlates of long-term e-cigarette and nicotine replacement therapy use: a prospective study in England

Sarah E. Jackson[1], Emily Hill[1], Lion Shahab[1], Emma Beard[1], Susan Michie[2], Jamie Brown[1,2]

[1] Department of Behavioural Science and Health, University College London, London, UK

[2] Department of Clinical, Educational and Health Psychology, University College London, UK

Key words: e-cigarettes; nicotine replacement therapy; population-based survey; long-term use.

Word count: 3,661

Number of tables: 3

Number of figures: 0

Corresponding author: Sarah E Jackson, PhD. Department of Behavioural Science and Health, University College London, 1-19 Torrington Place, London WC1E 6BT

Tel: (44) 207 679 3179

Fax: (44) 207 916 8354

s.e.jackson@ucl.ac.uk

For peer review only

Abstract

Objectives: To examine the prevalence of, and sociodemographic and smoking-related characteristics associated with, long-term e-cigarette use compared with long-term NRT use.

Design: Cross-sectional and prospective survey, the Smoking Toolkit Study, with baseline data collected between September 2014 and September 2016 and follow-ups at 6 and 12 months.

Setting: England.

Participants: Population-representative sample of 40,933 adults aged 16+.

Main outcome measures: Prevalence of long-term (≥12 months) use of e-cigarettes and NRT by retrospective self-report among baseline respondents (all adults, *n*=40,933; smokers, *n*=8,406), and current use at baseline, 6 months and 12 months in a subsample of smokers who responded to follow-up (*n*=733).

Results: Of baseline respondents, 1.5% (95%CI=1.4-1.6%, *n*=604) of adults and 3.9% (95%CI=3.5-4.3%, *n*=327) of smokers were long-term e-cigarette users and 0.5% (95%CI=0.4-0.6%, *n*=205) of adults and 1.3% (95%CI=1.1-1.5%, *n*=112) of smokers were long-term NRT users. Assessed prospectively, 13.4% (95%CI=10.9-15.9%, *n*=100) of smokers were long-term e-cigarette users and 1.9% (95%CI=0.9-2.9%, *n*=14) were long-term NRT users. Among all adults, long-term use by never smokers of either e-cigarettes (0.1%, *n*=27) or NRT (0.0%, *n*=7) was rare. Among past-year smokers, long-term e-cigarette and NRT use was higher among older smokers compared with 16-34 year-olds (OR range=1.55-5.21). Long-term e-cigarette use only was lower in smokers who were less educated (OR=0.63, 95%CI=0.49-0.81), from social grades C2DE (OR=0.66, 95%CI 0.52-0.84) and with children in the household (OR=0.66, 95%CI=0.51-0.85). Long-term e-cigarette use and long-term NRT use were higher among smokers more motivated to quit (OR=2.05, 95%CI=1.63-2.60 and OR=2.33, 95%CI=1.57-3.46).

Conclusions: In the adult population in England, long-term use of e-cigarettes and long-term use of NRT are almost exclusively by current or ex-smokers. Only a minority of past-year smokers retrospectively report long-term e-cigarette or NRT use but this figure may be an underestimate, especially for e-cigarette use, which is more than three-fold higher when assessed prospectively.

For peer review only - http://bmjopen.bmj.com/site/about/guidelines.xhtml

For peer review only

Strengths and limitations of the study

- Large sample representative of the adult population in England.
- Longitudinal design permitting prospective assessment of long-term use in addition to cross-sectional analyses based on retrospective self-reports.
- Only respondents who reported past-year smoking at baseline were invited to participate in the follow-up surveys, so we were unable to obtain prospective estimates of the prevalence of long-term e-cigarette or NRT use in the entire adult population.
- Substantial attrition bias meant our sample for prospective analyses was older and more socioeconomically advantaged than the group who were lost to follow-up, and more reported recent quitting and long-term use of e-cigarettes or NRT retrospectively at baseline.

For peer review only

Introduction

Tobacco smoking is one of the leading causes of premature death and disability worldwide (1). The primary cause of smoking-associated morbidity and mortality is the inhalation of toxins produced from the combustion of tobacco (2). Over recent years, electronic cigarettes (e-cigarettes) have rapidly become popular among smokers as a non-combustible alternative to cigarettes that offers safer nicotine delivery (3). But while the prevalence of ever and current use of e-cigarettes has been monitored (e.g., 4–6), there has been little investigation into long-term use of these products. Given an increasing focus on harm reduction in tobacco control, which aims to reduce the harm from combustible products by partial or complete substitution with non-combustible products, high-quality data on long-term use are needed. Understanding who is using e-cigarettes, and for how long, is fundamental in order to evaluate their overall impact on public health.

In England, e-cigarettes are used by around 5% of the adult population (~20% of smokers) (3) and have overtaken nicotine replacement therapy (NRT) as the most popular quitting aid, with over a third of smokers using an e-cigarette in their most recent quit attempt compared with one in five using of NRT (7). In England, e-cigarettes are not currently available on prescription but are subject to the EU Tobacco Products Directive (including advertising restrictions) and Trading Standards and can be bought online and from vape shops, pharmacies and other retail outlets, while NRT can be bought over the counter or obtained on prescription from a licensed health professional. Evidence from three randomised controlled trials indicates that using e-cigarettes in a quit attempt increases chances of successful cessation (8,9). On a population level, the rise in use of e-cigarettes in England and the US has been associated with increases in the overall success rate of quit attempts in the population (10,11), likely contributing to continued declines in smoking prevalence (12). It is possible that long-term e-cigarette use could help mitigate the high risk of relapse among recent quitters (13); in a survey of US smokers with two-year follow-up, long-term use of e-cigarettes (current use at baseline and follow-up) was associated with four times higher odds of cessation relative to no use (14).

Accumulating evidence demonstrates that using e-cigarettes is substantially less harmful than smoking (3). Toxicology testing has shown that while e-cigarettes can be used to obtain similar levels of nicotine to combustible cigarettes, switching to e-cigarettes can significantly reduce levels of measured carcinogens and toxins relative to smoking only combustible cigarettes, with differences observed within a matter of weeks (15–17). A more favourable toxicity profile has also been observed among long-term e-cigarette

For peer review only - http://bmjopen.bmj.com/site/about/guidelines.xhtml

users (≥6 months) compared with current cigarette smokers (18). However, surveys have indicated that around half of smokers inaccurately judge e-cigarettes to be more harmful than combustible cigarettes, about as harmful, or are unsure about the relative risk (19), which could discourage use.

Previous studies that have examined correlates of e-cigarette use have found that smokers who use e-cigarettes tend to be younger than non-users, smoke more heavily, and are more likely to have tried to quit in the past year (4–6). There is also some evidence that e-cigarette use is more prevalent among people with greater socioeconomic advantage (5,6), although the gap appears to have narrowed over recent years (20). However, there is a distinct lack of evidence on both the prevalence of long-term use and the profile of long-term users. This information is important for the evaluation of the overall public health impact of e-cigarettes, which requires specification of a wide variety of parameters beyond the safety of e-cigarettes and their effect on cessation, including the extent and characteristics of people who become long-term users (21).

The present study therefore aimed to examine the prevalence of, and sociodemographic and smoking-related characteristics associated with, long-term (≥12 months) e-cigarette use in England. We also analysed data on long-term NRT use as a case-control, in order to assess the extent to which the prevalence of long-term e-cigarette use and profile of long-term users are specific to e-cigarettes or apply more broadly to non-combustible nicotine products in general. Specifically, we aimed to answer the following research questions:

1. What proportion of adults in England retrospectively report using (i) e-cigarettes or (ii) NRT for at least one year?
2. What proportion of past-year smokers in England retrospectively report using (i) e-cigarettes or (ii) NRT for at least one year?
3. What proportion of past-year smokers in England report current use of (i) e-cigarettes or (ii) NRT at baseline and both 6 and 12-month follow-ups?
4. How do long-term users of e-cigarettes and NRT differ from non-users in their sociodemographic and smoking-related characteristics?

Method

Design and study population

For peer review only - http://bmjopen.bmj.com/site/about/guidelines.xhtml

Data were used from the Smoking Toolkit Study (STS), an ongoing monthly repeat cross-sectional survey of adults in England (22). Each month, a form of random location sampling is used to select a new sample of approximately 1,700 adults aged 16 years and older. Grouped output areas (containing ~300 households) are stratified by ACORN (socio-demographic) characteristics (http://www.caci.co.uk/acron/acornmap.asp) and region before being randomly selected for inclusion in an interviewers list. Interviewers then choose which houses within these areas are most likely to fulfil their quotas and conduct face-to-face computer-assisted interviews with one member per household. Comparisons of sociodemographic data and smoking prevalence and consumption estimates with national data indicate that STS data are broadly representative of the English population, having a similar composition to other large national surveys, such as the Health Survey for England (22). All participants provide fully informed consent prior to participation. In each wave, respondents complete a face-to-face computer-assisted survey with a trained interviewer. Respondents to the baseline survey between September 2014 and September 2016 who reported smoking in the past year were asked whether they were willing to be re-contacted, and those who agreed were followed up by telephone 6 and 12 months after the baseline interview. Up to 7 attempts were made to follow up each consenting participant. For the purpose of the present study, we aggregated data across survey waves. Cross-sectional analyses used data from all adults who responded to the baseline survey during this period, and the prospective analysis used data from respondents who reported past-year smoking at baseline and responded to both the 6 and 12-month follow-up surveys.

Patient and public involvement

The wider toolkit study has been discussed with a diverse patient and public involvement (PPI) group, and the authors regularly attend and present at meetings at which patients and public are included. Interaction and discussion at these events help to shape the broad research priorities and questions. There is also a mechanism for generalized input from the wider public: each month interviewers seek feedback on the questions from all 1700 respondents, who are representative of the English population. This feedback is limited, and usually simply relates to understanding of questions and item options. No patients or members of the public were involved in setting the research questions or the outcome measures, nor were they involved in the design and implementation of this specific study. There are no plans to involve patients in dissemination.

Measures

Outcomes: long-term use of e-cigarettes and NRT

For peer review only - http://bmjopen.bmj.com/site/about/guidelines.xhtml

The outcomes were long-term (≥12 months) use of e-cigarettes and long-term use of NRT, assessed retrospectively at baseline and prospectively over a 12-month follow-up.

In each of the baseline and follow-up surveys, three questions asked respondents about current use of e-cigarettes (or other vaping devices) and NRT (e.g. nicotine patches, gum, spray, or any other product):

1. Which, if any, of these are you currently using to help you cut down the amount you smoke?
2. Do you regularly use either of these in situations when you are not allowed to smoke?
3. Can I check, do you currently use either of the following at all for any reason?

In the baseline survey, respondents reporting use of either e-cigarettes or NRT were asked: "How long have you been using this nicotine replacement product or these products for?" Response options were: (i) less than one week, (ii) one to six weeks, (iii) more than six weeks and up to 12 weeks, (iv) more than 12 weeks and up to 26 weeks, (v) more than 26 weeks and up to 52 weeks, and (vi) more than 52 weeks.

For the present analyses, long-term use of e-cigarettes/NRT was defined as current use initiated more than 52 weeks prior to the baseline survey for cross-sectional analyses, and as current use at baseline, 6 months and 12 months for prospective analyses. Participants who reported long-term use of both e-cigarettes and NRT (*n*=66) were excluded.

Sociodemographic and smoking-related characteristics

Data were included on a range of sociodemographic and smoking-related characteristics assessed at baseline, selected *a priori* on the basis of previous studies demonstrating associations with use of e-cigarettes and/or NRT.

Sociodemographic information included: age, sex, ethnicity, region, social grade, level of education, disability, and the presence of children in the household. Ethnicity was categorised as white vs. non-white. Region was defined according to Government Office Region, grouped into three categories: northern, central and southern England. Social grade was categorised as ABC1 (which includes managerial, professional and intermediate occupations) vs. C2DE (which includes small employers and own-account workers, lower supervisory and technical occupations, and semi-routine and routine occupations, never workers and long-term unemployed). This occupational measure of social grade is a valid index of SES that is widely used in research in UK populations. It has been identified as particularly relevant in the context of tobacco use and quitting (23) and other addictive behaviours (24). These social grades are frequently

For peer review only - http://bmjopen.bmj.com/site/about/guidelines.xhtml

amalgamated into two groupings; ABC1 and C2DE. Here, researchers frequently interpret ABC1 to represent the middle class and C2DE to represent the working class. Education was categorised as lower (no post-16 qualifications) vs. higher (higher-level qualifications above GCSE level). Disability status was identified from the question “Do you consider yourself to have a disability within the meaning of the Disability Discrimination Act 1995 (yes/no)?”. The number of children in the household was self-reported and dichotomised to 0 vs. ≥1.

Smoking-related characteristics included: smoking status, time to first cigarette, consistent motivation to stop and (because it has been shown to be associated with smoking and quitting behaviour (25–27)) high-risk drinking. Smoking status was self-reported by all adults in response to the question: “Which of the following best applies to you (current smoker/stopped in the past year/stopped more than a year ago/never smoked)?”. Respondents who reported current smoking or having stopped in the past year (‘past-year smokers’) were also asked how soon after waking they typically smoked their first cigarette (categorised as within 30 vs. ≥31 minutes; an established indicator of nicotine dependence (28)) and whether they had consistently felt that they wanted to stop in the past year (yes/no). High-risk drinking was assessed using the Alcohol Use Disorders Identification Test (AUDIT) (29), a 10-item screening tool developed by the World Health Organisation to assess alcohol consumption, drinking behaviours and alcohol dependence, with a score of 8 or more indicating high-risk drinking.

Statistical analysis

The analysis plan and syntax were pre-registered on Open Science Framework (https://osf.io/bpjhk/). All analyses were done in SPSS v.25 on complete cases.

We used chi-square tests to compare the baseline characteristics of individuals who responded to both the 6 and 12-month follow-ups with those who were lost to follow-up in order to assess the representativeness of those followed-up.

We estimated the weighted prevalence of long-term use of (i) e-cigarettes and (ii) NRT in the total adult population at baseline, and in past-year smokers at baseline and over 12-month follow-up. Rim (marginal) weighting was used to match the English population on the dimensions of age, social grade, region, housing tenure (bought on a mortgage, owned outright, rented from local authority, rented from private landlord), ethnicity and working status (working, not working) within sex.

For peer review only - http://bmjopen.bmj.com/site/about/guidelines.xhtml

We then used logistic regression to examine the extent to which sociodemographic and smoking-related characteristics were associated with long-term use of e-cigarettes and NRT, assessed at baseline. For each outcome, we analysed bivariate associations with each potential correlate separately, and tested independent associations with a multivariable model that included all variables. We had also intended to analyse associations with long-term use prospectively, but the achieved sample size was lower than we had anticipated, and the prevalence of long-term use was low (particularly for NRT), limiting statistical power.

Following peer review, we added an unplanned sensitivity analysis of the prospective data in which missing data on e-cigarette and NRT use at 6 months and 12 months were imputed for all baseline past-year smokers with missing data. We used a multiple imputation model with all baseline sociodemographic and smoking-related characteristics, baseline use of e-cigarettes, and baseline use of NRT as predictors. Five imputed datasets were created, each analysed separately, and the results combined to produce pooled estimates of prevalence of long-term use of e-cigarettes and long-term use of NRT.

Results

Long-term use of e-cigarettes and NRT among all adults in England: cross-sectional data

A total of 42,040 adults in England were surveyed between September 2014 and September 2016 and 40,933 (97.4%) were complete cases. The weighted prevalence of long-term e-cigarette use assessed retrospectively among all adults in England was 1.5% (95%CI 1.4-1.6%) and of long-term NRT use was 0.5% (95%CI 0.4-0.6%). Table 1 summarises sample characteristics and bivariate and multivariable associations between sociodemographic and smoking-related characteristics and long-term use of e-cigarettes and NRT among all adults in the baseline survey.

In the multivariable model, both long-term e-cigarette use and long-term NRT use were significantly associated with age, region, level of education, disability, and smoking status. Compared with those aged 16-34, long-term e-cigarette use was more prevalent among those aged 35-54 but was not significantly different among those aged ≥55 years. Long-term NRT was significantly more prevalent among those aged 35-54 and ≥55 years. Compared with the north of England, long-term e-cigarette use was less prevalent in central and southern regions, and long-term NRT use was more prevalent in the south. Both long-term e-cigarette use and long-term NRT use were significantly less prevalent among people with no post-16

For peer review only - http://bmjopen.bmj.com/site/about/guidelines.xhtml

qualifications and more prevalent among those with a disability. Prevalence of long-term e-cigarette and NRT use did not differ significantly between current smokers and recent ex-smokers, but was significantly less prevalent among never smokers, among whom use of either product was rarely reported (e-cigarettes 0.1%, NRT 0.0%). Long-term NRT use was also significantly less prevalent among long-term ex-smokers, but the prevalence of long-term e-cigarette use did not differ significantly between long-term ex-smokers and current smokers.

Long-term NRT use, but not long-term e-cigarette use, was associated with sex and high-risk drinking, with higher prevalence observed among women and high-risk drinkers. Long-term e-cigarette use, but not long-term NRT use, was associated with the presence of children in the household, with lower prevalence observed among people with children in their household. We observed no significant association between long-term e-cigarette or NRT use and ethnicity or social grade.

Long-term use of e-cigarettes and NRT among past-year smokers in England: cross-sectional data

A total of 8,649 participants were past-year smokers and 8,406 (97.2%) were complete cases. The weighted prevalence of long-term e-cigarette use assessed retrospectively among past-year smokers in England was 3.9% (95%CI 3.5-4.3%) and of long-term NRT use was 1.3% (95%CI 1.1-1.5%). Table 2 summarises bivariate and multivariable associations between sociodemographic and smoking-related characteristics and long-term use of e-cigarettes and NRT among past-year smokers in the baseline survey.

In the multivariable model, there were significant associations between long-term use of e-cigarettes and NRT and age, region, and motivation to stop smoking, and between long-term use of e-cigarettes and social grade, level of education, and children in the household. Long-term use of e-cigarettes and NRT was more prevalent among older smokers compared with 16-34 year-olds, and among those who were motivated to stop. Compared with the north of England, long-term e-cigarette use was less prevalent in central and southern regions, but long-term NRT use was more prevalent in the south. Long-term e-cigarette use was significantly less prevalent among smokers from social grades C2DE, without post-16 qualifications and with children in their household, while long-term NRT use did not differ significantly according to these factors. We observed no significant association between long-term e-cigarette or NRT use and sex, ethnicity, disability, current smoking status, excessive drinking, or dependence.

Long-term use of e-cigarettes and NRT among all adults in England: prospective data

For peer review only - http://bmjopen.bmj.com/site/about/guidelines.xhtml

A total of 733 individuals who reported past-year smoking at baseline completed follow-up surveys at both 6 and 12 months. Characteristics of past-year smokers in the baseline and follow-up samples are summarised in Table 3. Past-year smokers who responded to follow-up were significantly older than those who did not. A higher proportion of responders were white and fewer were from social grades CD2E or had no post-16 qualifications. More reported a disability and fewer had children in the household. A higher proportion of responders than non-responders were recent ex-smokers and more reported consistent motivation to stop smoking. They were also significantly more likely to report long-term use of e-cigarettes or NRT than those who did not respond to the follow-up surveys. Loss to follow-up was not significantly associated with sex, region, high-risk drinking, or dependence. The weighted prevalence of long-term e-cigarette use assessed prospectively among past-year smokers in England was 13.4% (95%CI 10.9-15.9%) and of long-term NRT use was 1.9% (95%CI 0.9-2.9%).

When missing data on use of e-cigarettes and NRT at 6 and 12 months were multiply imputed for participants who were past-year smokers at baseline and did not participate in the follow-up surveys (*n*=1,673, 69.5% of all baseline past-year smokers), the unweighted prevalence of long-term e-cigarette use assessed prospectively was 9.8% (95%CI 9.2-10.4%) and of long-term NRT use was 1.7% (95%CI 1.4-2.0%), and the weighted prevalence was 10.3% (95%CI 9.7-11.0%) and 1.6% (95%CI 1.3-1.9%), respectively.

Discussion

In this large, representative sample of adults in England, long-term use of e-cigarettes and NRT was almost exclusively reported by current or ex-smokers. Only a minority of past-year smokers retrospectively reported long-term use of either e-cigarettes (3.9%) or NRT (1.3%) but this figure may be an underestimate: prevalence of current use at three time-points over a 12-month period was substantially higher for both e-cigarettes (13.4%) and NRT (1.9%), although these estimates were likely subject to attrition bias. When missing data were imputed, prospectively assessed prevalence estimates were slightly lower, at 10.3% for long-term e-cigarette use and 1.6% for long-term NRT use. Both cross-sectionally and prospectively, there was a higher prevalence of long-term e-cigarette use in comparison with NRT use. In adjusted models, long-term use of e-cigarettes and NRT was higher among older smokers and those more motivated to quit smoking. Long-term use of e-cigarettes was less common, and long-term use of NRT was more common, in the south of England compared with the north. Long-term use of e-cigarettes was significantly less prevalent

For peer review only - http://bmjopen.bmj.com/site/about/guidelines.xhtml

among smokers who were less educated, those from social grades C2DE, and those with children in the household, but these variables were not significantly associated with long-term use of NRT. Neither long-term use of e-cigarettes nor NRT differed significantly according to sex, ethnicity, disability, current smoking status (current vs. recent ex-smokers), high-risk drinking, or nicotine dependence.

To our knowledge, this study is the first to examine the prevalence of, and sociodemographic and smoking-related characteristics associated with, long-term use of e-cigarettes. We aimed to identify the prevalence of long-term e-cigarette use cross-sectionally and prospectively, and to contrast usage with long-term NRT use. Our results showed a higher prevalence of long-term e-cigarette use in comparison with NRT. Recent prevalence estimates indicate that current use of e-cigarettes is much more popular than NRT (7) and the same appears true for long-term use.

Long-term use of both products was almost exclusively observed among current and former smokers. Concerns have been raised that e-cigarettes may serve as a gateway to cigarette smoking among never-smokers, particularly among youth (30,31), but in our sample, the prevalence of long-term e-cigarette use among never-smokers was just 0.1%, comparable to long-term NRT use. As such, the potential number of people susceptible to any gateway effects as a result of long-term e-cigarette use in England between 2014 and 2016 appears to have been very small. We also observed higher prevalence of long-term e-cigarette use among middle-aged and older adults than in the youngest group (16-34 years), in contrast with evidence that current use of e-cigarettes among current and former smokers in England is least prevalent in the oldest age group (12.2% in those aged ≥65 years, compared with 18.7%, 21.4%, 20.8%, 20.6%, and 18.6% in those aged 16-24, 25-34, 35-44, 45-54, and 55-64 years) (7).

While the prevalence of long-term use of e-cigarettes and NRT did not differ significantly between current and recent (<1 year) ex-smokers, the relative prevalence of use in long-term (≥1 year) ex-smokers differed between the products. Long-term use of NRT was significantly less prevalent among long-term ex-smokers than current smokers, whereas long-term use of e-cigarettes was similar between these groups. This suggests that people tend to discontinue use of NRT more quickly after quitting smoking than with e-cigarettes, possibly because e-cigarettes are a closer substitute for the behaviour of cigarette smoking than NRT, or because NRT is viewed more as a medication than a recreational product (9). A recent trial of e-cigarettes compared with NRT in UK stop smoking services observed similar, with participants randomised to use e-cigarettes in a quit attempt more likely than those randomised to use NRT to still be using their allocated product one year later (80% vs. 9%, respectively) (9).

For peer review only - http://bmjopen.bmj.com/site/about/guidelines.xhtml

Insofar that use of alternative nicotine products should promote cessation rather than continued dual use, it is somewhat concerning that long-term use of e-cigarettes was similarly prevalent among current and recent ex-smokers. On the other hand, this appeared equally true for NRT. There have been concerns that dual use of cigarettes and e-cigarettes could reduce the urgency to quit smoking (32) and extend the duration of cigarette smoking (33,34). This would result in a negative overall public health impact, since duration of smoking poses a greater health risk than intensity of smoking (35). However, our results indicate that this is not the case: after mutual adjustment, the recall of long-term use of both e-cigarettes and NRT was higher among smokers who were more motivated to quit. This finding is consistent with previous studies showing that the most common reason for using an e-cigarette is to stop smoking (3), that smokers who use e-cigarettes are more likely to have recently tried to quit (4–6), and that long-term e-cigarette use is associated with a higher rate of smoking cessation (14). It suggests that long-term dual use is not associated with reduced motivation to quit.

Among past-year smokers, long-term use of e-cigarettes specifically was lower among those without post-16 qualifications and those from social grades C2DE. This is consistent with a larger literature on the diffusion of innovation, which recognises the tendency for high status groups to most quickly adopt new ideas and behaviours (36–38); a pattern that was documented for combustible cigarette smoking (39). According to this theory, one would expect to see e-cigarette use spread first within more affluent social networks, but patterns of imitation later lead to diffusion of the practice and normative change across the socio-economic range. The fact that we observed no significant association between education or social grade and long-term NRT use, which has a similar cost to users (40) but has been available for much longer, is consistent with this. With recent evidence indicating that the socioeconomic gradient in e-cigarette use is declining over time (20), we predict that this disparity in long-term use will disappear over the coming years. There were also regional differences, with long-term e-cigarette use more prevalent in the north of England and long-term NRT use more prevalent in the south. The higher prevalence of long-term e-cigarette use in the north is consistent with previous evidence of heavier smoking in the north of England (41) and higher prevalence of e-cigarette use among heavier smokers (4–6).

Prospective analysis of current use at baseline and both 6 and 12-month follow-ups indicated a substantially higher rate of long-term use of both e-cigarettes (13.4%) and NRT (1.9%) among past-year smokers than was seen in the cross-sectional results. While these figures are not directly comparable given the substantial attrition over follow-up assessments and differences in the definition of long-term use (continued use vs. current use at three defined time points), the magnitude of the difference indicates that retrospective recall

For peer review only - http://bmjopen.bmj.com/site/about/guidelines.xhtml

of how long the products have been used may underestimate what could be expected if users were followed more frequently over time. A study with a greater number of follow-up points over a longer period could offer further insight into this discrepancy.

Strengths of this study include the large, representative sample and prospective design. However, there were several limitations. Only respondents who reported past-year smoking at baseline were invited to participate in the follow-up surveys, so we were unable to obtain prospective estimates of the prevalence of long-term e-cigarette or NRT use in the entire adult population. While, evidence from the cross-sectional results of this study and from previous research (3) suggest that the vast majority of long-term users were current or recent ex-smokers, with low prevalence among never smokers, it would have been useful to have data from long-term ex-smokers. Another potential issue was substantial attrition bias. Our sample for prospective analyses was older and more socioeconomically advantaged than the group who were lost to follow-up, and more reported long-term use of e-cigarettes or NRT retrospectively at baseline. They were also more likely to have quit recently. This may have contributed to the higher prevalence of long-term use observed in prospective analyses. Finally, we did not consider reasons for or patterns of use. Future studies could build on our findings through more detailed or frequent assessments, and qualitative work with long-term users.

Conclusions

Long-term e-cigarette use is more prevalent than long-term NRT use in the English adult population, but both are almost exclusively by current or ex-smokers. The profile of long-term e-cigarette users is broadly similar to that of long-term NRT users, although there are some sociodemographic and regional differences between the two, with long-term e-cigarette use more prevalent among smokers with greater socioeconomic advantage and in the north of England, and long-term NRT use more prevalent among smokers in the south. Prospective assessment of long-term use produces substantially higher estimates of prevalence, particularly for e-cigarettes, than retrospective recall, although this may to some extent be accounted for by differences in the sample and definitions used. These results add to the descriptive picture of e-cigarette use in England, providing novel insight into long-term use. This information can be incorporated into broader evaluations of population-level use of e-cigarettes and their potential impact on public health.

For peer review only - http://bmjopen.bmj.com/site/about/guidelines.xhtml

References

1. World Health Organization. Tobacco [Internet]. 2018 [cited 2018 Dec 2]. Available from: http://www.who.int/news-room/fact-sheets/detail/tobacco

2. Royal College of Physicians. Harm reduction in nicotine addiction: helping people who can’t quit [Internet]. 2007 [cited 2018 Dec 5]. Available from: https://shop.rcplondon.ac.uk/products/harm-reduction-in-nicotine-addiction-helping-people-who-cant-quit

3. McNeill A, Brose LS, Calder R, Bauld L, Robson D. Evidence review of e-cigarettes and heated tobacco products 2018. A report commissioned by Public Health England. [Internet]. 2018 [cited 2018 Jun 21]. Available from: https://www.gov.uk/government/publications/e-cigarettes-and-heated-tobacco-products-evidence-review/evidence-review-of-e-cigarettes-and-heated-tobacco-products-2018-executive-summary

4. Vardavas CI, Filippidis FT, Agaku IT. Determinants and prevalence of e-cigarette use throughout the European Union: a secondary analysis of 26 566 youth and adults from 27 Countries. Tob Control. 2015 Sep 1;24(5):442–8.

5. Levy DT, Yuan Z, Li Y. The Prevalence and Characteristics of E-Cigarette Users in the U.S. Int J Environ Res Public Health [Internet]. 2017 Oct [cited 2018 Dec 5];14(10). Available from: https://www.ncbi.nlm.nih.gov/pmc/articles/PMC5664701/

6. Brown J, West R, Beard E, Michie S, Shahab L, McNeill A. Prevalence and characteristics of e-cigarette users in Great Britain: Findings from a general population survey of smokers. Addict Behav. 2014 Jun 1;39(6):1120–5.

7. West R, Proudfoot H, Beard E, Brown J. Electronic cigarettes in England - latest trends [Internet]. Smoking in England; 2019 [cited 2019 May 26]. Available from: http://www.smokinginengland.info/latest-statistics/

8. McRobbie H, Bullen C, Hartmann-Boyce J, Hajek P. Electronic cigarettes for smoking cessation and reduction. Cochrane Database Syst Rev. 2014;(12):CD010216.

9. Hajek P, Phillips-Waller A, Przulj D, Pesola F, Myers Smith K, Bisal N, et al. A Randomized Trial of E-Cigarettes versus Nicotine-Replacement Therapy. N Engl J Med. 2019 Feb 14;380(7):629–37.

10. Beard E, West R, Michie S, Brown J. Association between electronic cigarette use and changes in quit attempts, success of quit attempts, use of smoking cessation pharmacotherapy, and use of stop smoking services in England: time series analysis of population trends. BMJ. 2016 Sep 13;354:i4645.

11. Zhu S-H, Zhuang Y-L, Wong S, Cummins SE, Tedeschi GJ. E-cigarette use and associated changes in population smoking cessation: evidence from US current population surveys. BMJ. 2017 Jul 26;358:j3262.

12. Royal College of Physicians. Nicotine without smoke: Tobacco harm reduction [Internet]. 2016 Apr [cited 2018 Dec 5]. Available from: https://www.rcplondon.ac.uk/projects/outputs/nicotine-without-smoke-tobacco-harm-reduction-0

For peer review only

For peer review only - http://bmjopen.bmj.com/site/about/guidelines.xhtml

13. Hughes JR, Keely J, Naud S. Shape of the relapse curve and long-term abstinence among untreated smokers. Addict Abingdon Engl. 2004 Jan;99(1):29–38.

14. Zhuang Y-L, Cummins SE, Sun JY, Zhu S-H. Long-term e-cigarette use and smoking cessation: a longitudinal study with US population. Tob Control. 2016 Oct 1;25(Suppl 1):i90–5.

15. Goniewicz ML, Gawron M, Smith DM, Peng M, Jacob P, Benowitz NL. Exposure to Nicotine and Selected Toxicants in Cigarette Smokers Who Switched to Electronic Cigarettes: A Longitudinal Within-Subjects Observational Study. Nicotine Tob Res. 2017 Feb 1;19(2):160–7.

16. McRobbie H, Phillips A, Goniewicz ML, Smith KM, Knight-West O, Przulj D, et al. Effects of Switching to Electronic Cigarettes with and without Concurrent Smoking on Exposure to Nicotine, Carbon Monoxide, and Acrolein. Cancer Prev Res Phila Pa. 2015 Sep;8(9):873–8.

17. Hecht SS, Carmella SG, Kotandeniya D, Pillsbury ME, Chen M, Ransom BWS, et al. Evaluation of toxicant and carcinogen metabolites in the urine of e-cigarette users versus cigarette smokers. Nicotine Tob Res Off J Soc Res Nicotine Tob. 2015 Jun;17(6):704–9.

18. Shahab L, Goniewicz ML, Blount BC, Brown J, McNeill A, Alwis KU, et al. Nicotine, Carcinogen, and Toxin Exposure in Long-Term E-Cigarette and Nicotine Replacement Therapy Users: A Cross-sectional Study. Ann Intern Med. 2017 Mar 21;166(6):390–400.

19. Wilson S, Partos T, McNeill A, Brose LS. Harm perceptions of e-cigarettes and other nicotine products in a UK sample. Addict Abingdon Engl. 2019 Jan 3;

20. Kock L, Shahab L, West R, Brown J. E-cigarette use in England 2014-17 as a function of socio-economic profile. Addict Abingdon Engl. 2018 Oct 10;

21. Levy DT, Cummings KM, Villanti AC, Niaura R, Abrams DB, Fong GT, et al. A framework for evaluating the public health impact of e-cigarettes and other vaporized nicotine products. Addict Abingdon Engl. 2017;112(1):8–17.

22. Fidler JA, Shahab L, West O, Jarvis MJ, McEwen A, Stapleton JA, et al. “The smoking toolkit study”: a national study of smoking and smoking cessation in England. BMC Public Health. 2011 Jun 18;11:479.

23. Kotz D, West R. Explaining the social gradient in smoking cessation: it’s not in the trying, but in the succeeding. Tob Control. 2009 Feb;18(1):43–6.

24. Beard E, Brown J, West R, Kaner E, Meier P, Michie S. Associations between socio-economic factors and alcohol consumption: A population survey of adults in England. PLOS ONE. 2019 Feb 4;14(2):e0209442.

25. Falk DE, Yi H, Hiller-Sturmhöfel S. An epidemiologic analysis of co-occurring alcohol and tobacco use and disorders: findings from the National Epidemiologic Survey on Alcohol and Related Conditions. Alcohol Res Health J Natl Inst Alcohol Abuse Alcohol. 2006;29(3):162–71.

26. Beard E, West R, Michie S, Brown J. Association between smoking and alcohol-related behaviours: a time–series analysis of population trends in England. Addict Abingdon Engl. 2017 Oct;112(10):1832–41.

For peer review only

For peer review only - http://bmjopen.bmj.com/site/about/guidelines.xhtml

27. Brown J, West R, Beard E, Brennan A, Drummond C, Gillespie D, et al. Are recent attempts to quit smoking associated with reduced drinking in England? A cross-sectional population survey. BMC Public Health. 2016 Jul 22;16(1):535.

28. Heatherton TF, Kozlowski LT, Frecker RC, FAGERSTROM K-O. The Fagerström test for nicotine dependence: a revision of the Fagerstrom Tolerance Questionnaire. Br J Addict. 1991;86(9):1119–1127.

29. Babor TF, Higgins-Biddle JC, Saunders JB, Monteiro MG, Organization WH. AUDIT: the alcohol use disorders identification test: guidelines for use in primary health care. 2001;

30. Soneji S, Barrington-Trimis JL, Wills TA, Leventhal AM, Unger JB, Gibson LA, et al. Association Between Initial Use of e-Cigarettes and Subsequent Cigarette Smoking Among Adolescents and Young Adults: A Systematic Review and Meta-analysis. JAMA Pediatr. 2017 Aug 1;171(8):788–97.

31. Schneider S, Diehl K. Vaping as a Catalyst for Smoking? An Initial Model on the Initiation of Electronic Cigarette Use and the Transition to Tobacco Smoking Among Adolescents. Nicotine Tob Res Off J Soc Res Nicotine Tob. 2016 May;18(5):647–53.

32. Grana R, Benowitz N, Glantz SA. E-cigarettes: a scientific review. Circulation. 2014 May 13;129(19):1972–86.

33. Furlow B. US Government warns against long-term dual use of conventional and e-cigarettes. Lancet Respir Med. 2015 May;3(5):345.

34. Maziak W. Harm reduction at the crossroads: the case of e-cigarettes. Am J Prev Med. 2014 Oct;47(4):505–7.

35. Doll R, Peto R. Cigarette smoking and bronchial carcinoma: dose and time relationships among regular smokers and lifelong non-smokers. J Epidemiol Community Health. 1978 Dec 1;32(4):303–13.

36. Strang D, Soule SA. Diffusion in Organizations and Social Movements: From Hybrid Corn to Poison Pills. Annu Rev Sociol. 1998;24(1):265–90.

37. Rogers EM. Diffusion of Innovations, 4th Edition. Simon and Schuster; 2010. 550 p.

38. Katz E. Theorizing Diffusion: Tarde and Sorokin Revisited. Ann Am Acad Pol Soc Sci. 1999 Nov 1;566(1):144–55.

39. Ferrence R. Deadly Fashion: The Rise and Fall of Cigarette Smoking in North America. 1 edition. New York: Garland Science; 1990. 167 p.

40. Jackson SE, Shahab L, Kock L, West R, Brown J. Expenditure on smoking and alternative nicotine delivery products: a population survey in England. Addiction. 2019;

41. Beard E, Brown J, West R, Angus C, Kaner E, Michie S. Healthier central England or North–South divide? Analysis of national survey data on smoking and high-risk drinking. BMJ Open. 2017 Mar 1;7(3):e014210.

For peer review only

For peer review only

Declarations

Ethics approval and consent to participate

Ethical approval for the Smoking Toolkit Study was granted originally by the UCL Ethics Committee (ID 0498/001) and participants provided full informed consent. The data are not collected by UCL and are anonymised when received by UCL.

Availability of data and materials

The datasets used and/or analysed during the current study are available from the corresponding author on reasonable request.

Competing interests

JB received unrestricted research funding from Pfizer, who manufacture smoking cessation medications. LS has received honoraria for talks, an unrestricted research grant and travel expenses to attend meetings and workshops from Pfizer, and has acted as paid reviewer for grant awarding bodies and as a paid consultant for health care companies. All authors declare no financial links with tobacco companies or e-cigarette manufacturers or their representatives.

Funding

This work was supported by Cancer Research UK, grant numbers C1417/A22962 and C44576/A19501. The funders had no final role in the study design; in the collection, analysis and interpretation of data; in the writing of the report; or in the decision to submit the paper for publication. All researchers listed as authors are independent from the funders and all final decisions about the research were taken by the investigators and were unrestricted.

Author contributions

JB and EH conceived of the study. SEJ, EH and JB analysed and interpreted the data. SEJ and EH drafted the manuscript. SEJ, EH, LS, EB, SM and JB critically revised the manuscript for important intellectual content.

For peer review only

Table 1 Sample descriptive characteristics and factors associated with long-term use of e-cigarettes or NRT by adults in England: cross-sectional da
(*n*=40,933)

	Whole sample	E-cigarettes			NRT		
	% (*n*)	% (*n*) long-term use	OR [95% CI] *p*	Adj OR [95% CI] *p*	% (*n*) long-term use	OR [95% CI] *p*	Adj O
All adults*	-	1.5 (604)	-	-	0.5 (205)	-	
Age in years							
16-34	30.3 (12,398)	1.1 (138)	1.00	1.00	0.2 (21)	1.00	
35-54	29.6 (12,118)	2.1 (252)	1.89 (1.53:2.33) <0.001	1.71 (1.38:2.13) <0.001	0.7 (87)	4.26 (2.65:6.87) <0.001	3.98
≥55	40.1 (16,417)	1.1 (185)	1.01 (0.81:1.26) 0.912	0.78 (0.60:1.02) 0.064	0.6 (89)	3.21 (2.00:5.17) <0.001	3.25
Sex							
Men	50.9 (20.816)	1.5 (308)	1.00	1.00	0.5 (94)	1.00	
Women	49.1 (20.117)	1.3 (267)	0.90 (0.76:1.06) 0.190	1.04 (0.87:1.23) 0.683	0.5 (103)	1.14 (0.86:1.50) 0.377	1.39
Ethnicity							
Non-white	16.4 (6,730)	0.6 (42)	1.00	1.00	0.1 (10)	1.00	
White	83.6 (34,203)	1.6 (533)	2.52 (1.84:3.46) <0.001	1.12 (0.80:1.56) 0.510	0.5 (187)	3.69 (1.95:6.98) <0.001	1.48
Social grade							
ABC1	53.5 (21,894)	1.3 (292)	1.00	1.00	0.5 (102)	1.00	
C2DE	46.5 (19,039)	1.5 (283)	1.12 (0.95:1.32) 0.191	0.91 (0.76:1.10) 0.326	0.5 (95)	1.07 (0.81:1.42) 0.629	0.93
Region							
North	32.0 (13,111)	2.1 (281)	1.00	1.00	0.4 (57)	1.00	
Central	29.3 (12,000)	1.1 (132)	0.51 (0.41:0.63) <0.001	0.60 (0.48:0.74) <0.001	0.5 (59)	1.13 (0.79:1.63) 0.507	1.41
South	38.7 (15,822)	1.0 (162)	0.47 (0.39:0.57) <0.001	0.55 (0.45:0.67) <0.001	0.5 (81)	1.18 (0.84:1.66) 0.343	1.45
Level of education							
Post-16 qualifications	63.4 (25,945)	1.4 (371)	1.00	1.00	0.5 (126)	1.00	
No post-16 qualifications	36.6 (14,988)	1.4 (204)	0.95 (0.80:1.13) 0.569	0.79 (0.65:0.95) 0.012	0.5 (71)	0.98 (0.73:1.31) 0.867	0.71
Disability							
No	88.2 (36,119)	1.3 (463)	1.00	1.00	0.4 (152)	1.00	
Yes	11.8 (4,814)	2.3 (112)	1.83 (1.49:2.26) <0.001	1.34 (1.07:1.66) 0.009	0.9 (45)	2.23 (1.60:3.12) <0.001	1.52
Children in the household							
0	71.0 (29,062)	1.4 (413)	1.00	1.00	0.5 (145)	1.00	

For peer review only - http://bmjopen.bmj.com/site/about/guidelines.xhtml

≥1	29.0 (11,871)	1.4 (162)	0.96 (0.80:1.15) 0.660	0.80 (0.65:0.98) 0.030	0.4 (52)	0.88 (0.64:1.21) 0.420	1.00
Current smoking status							
Current smoker	19.3 (7,900)	3.8 (302)	1.00	1.00	1.4 (107)	1.00	
Recent (<1y) ex-smoker	1.4 (558)	3.8 (21)	0.98 (0.63:1.54) 0.944	0.93 (0.59:1.46) 0.747	1.3 (7)	0.93 (0.43:2.00) 0.843	0.87
Long-term (≥1y) ex-smoker	17.1 (7,009)	3.2 (225)	0.83 (0.70:1.00) 0.043	0.90 (0.74:1.09) 0.274	1.1 (76)	0.80 (0.59:1.07) 0.136	0.62
Never smoker	62.2 (25,466)	0.1 (27)	0.03 (0.02:0.04) <0.001	0.03 (0.02:0.04) <0.001	0.0 (7)	0.02 (0.01:0.04) <0.001	0.02
High-risk drinking							
No	87.3 (35,742)	1.3 (449)	1.00	1.00	0.4 (149)	1.00	
Yes	12,7 (5,191)	2.4 (126)	1.95 (1.60:2.39) <0.001	1.02 (0.83:1.27) 0.830	0.9 (48)	2.22 (1.61:3.09) <0.001	1.60

The adjusted model includes all variables in the table and year of survey.

*This figure is weighted, and therefore the effective N does not correspond precisely with the unweighted figures reported elsewhere in the table.

OR = odds ratio; CI = confidence interval; SD = standard deviation; NRT = nicotine replacement therapy.

For peer review only

For peer review only - http://bmjopen.bmj.com/site/about/guidelines.xhtml

Table 2 Factors associated with long-term use of e-cigarettes or NRT by past-year smokers in England: cross-sectional data (*n*=8,406)

	E-cigarettes			NRT		
	**% (*n*) long-term use**	**OR [95% CI] *p***	**Adj OR [95% CI] *p***	**% (*n*) long-term use**	**OR [95% CI] *p***	**Adj OR [95% CI] *p***
All adults*	3.9 (327)	-	-	1.3 (112)	-	-
Age in years						
16-34	2.6 (88)	1.00	1.00	0.5 (15)	1.00	1.00
35-54	4.9 (135)	1.88 (1.43:2.47) <0.001	1.90 (1.44:2.52) <0.001	1.7 (46)	3.72 (2.07:6.67) <0.001	3.47 (1.92:6.27) <0.001
≥55	4.3 (98)	1.64 (1.22:2.19) 0.001	1.55 (1.12:2.13) 0.008	2.3 (52)	5.10 (2.86:9.08) <0.001	5.21 (2.79:9.72) <0.001
Sex						
Men	1.3 (57)	1.00	1.00	1.3 (57)	1.00	1.00
Women	1.5 (56)	0.96 (0.77:1.20) 0.702	1.00 (0.80:1.27) 0.974	1.4 (56)	1.15 (0.79:1.66) 0.474	1.14 (0.78:1.68) 0.498
Ethnicity						
Non-white	3.4 (31)	1.00	1.00	0.9 (8)	1.00	1.00
White	3.9 (290)	1.15 (0.79:1.68) 0.460	1.01 (0.68:1.50) 0.961	1.4 (105)	1.62 (0.79:3.33) 0.192	1.33 (0.63:2.80) 0.459
Social grade						
ABC1	5.2 (167)	1.00	1.00		1.00	1.00
C2DE	3.0 (154)	0.56 (0.45:0.70) <0.001	0.66 (0.52:0.84) 0.001	1.1 (58)	0.65 (0.45:0.94) 0.022	0.71 (0.47:1.06) 0.091
Region						
North	4.7 (147)	1.00	1.00	1.0 (32)	1.00	1.00
Central	3.4 (81)	0.73 (0.55:0.96) 0.023	0.79 (0.59:1.04) 0.094	1.4 (33)	1.38 (0.85:2.25) 0.197	1.52 (0.92:2.50) 0.100
South	3.2 (93)	0.68 (0.52:0.88) 0.004	0.68 (0.52:0.90) 0.006	1.7 (48)	1.64 (1.05:2.58) 0.030	1.76 (1.11:2.79) 0.016
Level of education						
Post-16 qualifications	4.7 (214)	1.00	1.00	1.4 (65)	1.00	1.00
No post-16 qualifications	2.7 (107)	0.57 (0.45:0.72) <0.001	0.63 (0.49:0.81) <0.001	1.2 (48)	0.85 (0.59:1.24) 0.411	0.85 (0.56:1.27) 0.426
Disability						
No	3.7 (260)	1.00	1.00	1.2 (86)	1.00	1.00
Yes	4.7 (61)	1.29 (0.97:1.72) 0.079	1.22 (0.90:1.64) 0.197	2.1 (27)	1.73 (1.12:2.67) 0.014	1.46 (0.93:2.29) 0.103
Children in the household						
0	4.3 (240)	1.00	1.00	1.5 (83)	1.00	1.00
≥1	2.9 (81)	0.66 (0.51:0.85) 0.001	0.68 (0.51:0.90) 0.007	1.1 (30)	0.71 (0.47:1.08) 0.111	1.07 (0.66:1.72) 0.795
Current smoking status						
Current smoker	3.8 (301)	1.00	1.00	1.3 (106)	1.00	1.00
Recent (<1y) ex-smoker	3.7 (20)	0.96 (0.61:1.52) 0.858	0.69 (0.43:1.11) 0.124	1.3 (7)	0.95 (0.44:2.06) 0.904	0.69 (0.32:1.52) 0.361
High-risk drinking						

For peer review only

For peer review only - http://bmjopen.bmj.com/site/about/guidelines.xhtml

No	3.7 (242)	1.00	1.00	1.3 (84)	1.00	1.00
Yes	4.2 (79)	1.14 (0.88:1.48) 0.323	1.02 (0.78:1.35) 0.871	1.5 (29)	1.20 (0.79:1.84) 0.396	1.52 (0.97:2.38) 0.065
Time to first cigarette						
31 or more minutes	3.8 (163)	1.00	1.00	1.2 (53)	1.00	1.00
Within 30 minutes	3.9 (158)	1.02 (0.82:1.28) 0.833	1.07 (0.85:1.35) 0.581	1.5 (60)	1.20 (0.83:1.74) 0.341	1.16 (0.79:1.70) 0.447
Consistent motivation to stop						
No	2.6 (123)	1.00	1.00	0.9 (41)	1.00	1.00
Yes	5.4 (198)	2.11 (1.68:2.66) <0.001	2.05 (1.63:2.60) <0.001	1.9 (72)	2.27 (1.54:3.33) <0.001	2.33 (1.57:3.46) <0.001

The adjusted model includes all variables in the table and year of survey.
*This figure is weighted, and therefore the effective N does not correspond precisely with the unweighted figures reported elsewhere in the table.
OR = odds ratio; CI = confidence interval; SD = standard deviation; NRT = nicotine replacement therapy.

For peer review only - http://bmjopen.bmj.com/site/about/guidelines.xhtml

Table 3 Comparison of the baseline and follow-up samples of past-year smokers

	Baseline sample* % (*n*=8,406)	Follow-up sample % (*n*=733)	*p***
Age in years			
16-34	39.6 (3326)	15.6 (114)	<0.001
35-54	33.0 (2777)	34.4 (252)	-
≥55	27.4 (2303)	50.1 (367)	-
Women	46.2 (3885)	44.9 (329)	0.449
White ethnicity	89.1 (7488)	95.4 (699)	<0.001
Social grade C2DE	61.8 (5193)	51.3 (376)	<0.001
Region			
North	37.5 (3150)	39.4 (289)	0.137
Central	28.1 (2363)	25.0 (183)	-
South	34.4 (2893)	35.6 (261)	-
No post-16 qualifications	46.3 (3893)	40.4 (296)	0.001
Has a disability	15.5 (1304)	22.6 (166)	<0.001
≥1 children in the household	33.6 (2826)	22.9 (168)	<0.001
Current smoking status			
Current smoker	93.5 (7862)	91.7 (672)	0.033
Recent (<1y) ex-smoker	6.5 (544)	8.3 (61)	-
High-risk drinking	22.4 (1879)	20.1 (147)	0.118
First cigarette within 30 minutes	48.6 (4089)	50.3 (369)	0.336
Consistent motivation to stop	43.9 (3694)	48.6 (356)	0.008
Long-term e-cigarette use***	3.8 (321)	5.5 (40)	0.015
Long-term NRT use***	1.3 (113)	2.6 (19)	0.002

*Past-year smokers only.

**Comparison of respondents who did and did not provide follow-up data.

***Assessed at baseline.

For peer review only

STROBE Statement—Checklist of items that should be included in reports of ***cross-sectional studies***

	Item No	Recommendation	Page
**Title and abstract**	1	(*a*) Indicate the study's design with a commonly used term in the title or the abstract	1
		(*b*) Provide in the abstract an informative and balanced summary of what was done and what was found	2
**Introduction**			
Background/rationale	2	Explain the scientific background and rationale for the investigation being reported	4
Objectives	3	State specific objectives, including any prespecified hypotheses	5
**Methods**			
Study design	4	Present key elements of study design early in the paper	5
Setting	5	Describe the setting, locations, and relevant dates, including periods of recruitment, exposure, follow-up, and data collection	6
Participants	6	(*a*) Give the eligibility criteria, and the sources and methods of selection of participants	6
Variables	7	Clearly define all outcomes, exposures, predictors, potential confounders, and effect modifiers. Give diagnostic criteria, if applicable	6/7
Data sources/ measurement	8*	For each variable of interest, give sources of data and details of methods of assessment (measurement). Describe comparability of assessment methods if there is more than one group	6
Bias	9	Describe any efforts to address potential sources of bias	7
Study size	10	Explain how the study size was arrived at	8
Quantitative variables	11	Explain how quantitative variables were handled in the analyses. If applicable, describe which groupings were chosen and why	6/7
Statistical methods	12	(*a*) Describe all statistical methods, including those used to control for confounding	7/8
		(*b*) Describe any methods used to examine subgroups and interactions	7/8
		(*c*) Explain how missing data were addressed	8
		(*d*) If applicable, describe analytical methods taking account of sampling strategy	7/8
		(*e*) Describe any sensitivity analyses	na
**Results**			
Participants	13*	(a) Report numbers of individuals at each stage of study—eg numbers potentially eligible, examined for eligibility, confirmed eligible, included in the study, completing follow-up, and analysed	8
		(b) Give reasons for non-participation at each stage	8
		(c) Consider use of a flow diagram	na
Descriptive data	14*	(a) Give characteristics of study participants (eg demographic, clinical, social) and information on exposures and potential confounders	8/9
		(b) Indicate number of participants with missing data for each variable of interest	na
Outcome data	15*	Report numbers of outcome events or summary measures	Tables
Main results	16	(*a*) Give unadjusted estimates and, if applicable, confounder-adjusted estimates and their precision (eg, 95% confidence interval). Make clear which confounders were adjusted for and why they were included	Tables

For peer review only - http://bmjopen.bmj.com/site/about/guidelines.xhtml

For peer review only

		(*b*) Report category boundaries when continuous variables were categorized	8
		(*c*) If relevant, consider translating estimates of relative risk into absolute risk for a meaningful time period	na
Other analyses	17	Report other analyses done—eg analyses of subgroups and interactions, and sensitivity analyses	na
**Discussion**			
Key results	18	Summarise key results with reference to study objectives	10
Limitations	19	Discuss limitations of the study, taking into account sources of potential bias or imprecision. Discuss both direction and magnitude of any potential bias	12
Interpretation	20	Give a cautious overall interpretation of results considering objectives, limitations, multiplicity of analyses, results from similar studies, and other relevant evidence	13
Generalisability	21	Discuss the generalisability (external validity) of the study results	12
**Other information**			
Funding	22	Give the source of funding and the role of the funders for the present study and, if applicable, for the original study on which the present article is based	17

*Give information separately for exposed and unexposed groups.

Note: An Explanation and Elaboration article discusses each checklist item and gives methodological background and published examples of transparent reporting. The STROBE checklist is best used in conjunction with this article (freely available on the Web sites of PLoS Medicine at http://www.plosmedicine.org/, Annals of Internal Medicine at http://www.annals.org/, and Epidemiology at http://www.epidem.com/). Information on the STROBE Initiative is available at www.strobe-statement.org.

For peer review only

For peer review only - http://bmjopen.bmj.com/site/about/guidelines.xhtml
